# Glucocorticoids trigger muscle-liver crosstalk to attenuate acute liver injury and promote liver regeneration via the FGF6-FGFBP1 axis

**DOI:** 10.1186/s40779-025-00618-y

**Published:** 2025-07-21

**Authors:** Yue-Jie Xu, Cai-Zhi Liu, Ying Chen, Lan-Xin Li, Bo Xu, Ling-Xin You, Mei-Yao Meng, Xin Li, Hong Zhang, Qiu-Rong Ding, Rong Zhang, Xin-Ran Ma, Xiao-Hua Chen, Cheng Hu

**Affiliations:** 1 Department of Endocrinology and Metabolism, Shanghai Diabetes Institute, Shanghai Clinical Center for Diabetes, Shanghai Key Laboratory of Diabetes Mellitus, Shanghai Sixth People’s Hospital Affiliated to Shanghai Jiao Tong University School of Medicine, Shanghai, 200233 China; 2 Jinzhou Medical University Graduate Training Base (Shanghai Sixth People’s Hospital Affiliated to Shanghai Jiao Tong University School of Medicine), Jinzhou, 121001 Liaoning China; 3 Shanghai Key Laboratory of Regulatory Biology, Institute of Biomedical Sciences and School of Life Sciences, East China Normal University, Shanghai, 200241 China; 4 Research Direction of New Microecological Technology, Jinan Microecological Biomedicine Shandong Laboratory, Jinan, 250000 China; 5 CAS Key Laboratory of Nutrition, Metabolism and Food Safety, Shanghai Institute of Nutrition and Health, University of Chinese Academy of Sciences, Chinese Academy of Sciences, Shanghai, 200031 China; 6 Department of Infectious Diseases, Shanghai Sixth People’s Hospital Affiliated to Shanghai Jiao Tong University School of Medicine, Shanghai, 200233 China; 7 Department of Endocrinology and Metabolism, Fengxian Central Hospital Affiliated to Southern Medical University, Shanghai, 201499 China

**Keywords:** Acute liver injury (ALI), Liver regeneration, Skeletal muscle, Fibroblast growth factor 6 (FGF6), Fibroblast growth factor binding protein 1 (FGFBP1)

## Abstract

**Background:**

Acute liver injury (ALI) requires rapid hepatic regeneration to avert fatal liver failure. As key mechanisms, systemic metabolic remodeling and inter-organ crosstalk are critical for this regenerative process. Skeletal muscle, as a major metabolic organ system, undergoes significant remodeling during ALI. However, its specific regulatory contributions remain largely uncharacterized.

**Methods:**

Partial (2/3) hepatectomy and acetaminophen were used to induce ALI in male mice. RNA-sequencing (RNA-seq), assay for transposase-accessible chromatin by sequencing (ATAC-seq), chromatin immunoprecipitation, luciferase assay, Western blotting, TUNEL assay, immunohistochemistry, and phase separation assays were performed to reveal the transcriptional axis involved. Serum fibroblast growth factor binding protein 1 (FGFBP1) protein levels in ALI patients were assessed via enzyme-linked immunosorbent assay.

**Results:**

Integrated analysis of RNA-seq and ATAC-seq following ALI identifies glucocorticoid (GC) signaling-mediated regulation of fibroblast growth factor 6 (FGF6) in skeletal muscle metabolism. Muscle-specific knockdown of GC receptor (GR) exacerbates ALI and suppresses liver regeneration. *Fgf6*-knockout mice exhibited improved ALI and enhanced liver regeneration, with intramuscular injection of FGF6-neutralizing antibody rescuing the detrimental effects induced by GR knockdown. Further analysis of the FGF6 downstream target revealed that FGF6 regulates FGFBP1 expression through extracellular signal regulated kinase-activating transcription factor 3 signaling. Moreover, FGF6 regulates the heparin-dependent release kinetics of FGFBP1 by perturbing its liquid–liquid phase separation (LLPS)-driven condensate dynamics at the plasma membrane. Circulating FGFBP1 subsequently interacts with hepatic fibroblast growth factor 5 (FGF5) through LLPS mechanisms to regulate liver regeneration.

**Conclusion:**

Our results demonstrate a molecular mechanism by which muscle-liver crosstalk can initiate and sustain liver regeneration via the FGF6-FGFBP1/FGF5 axis, providing a potential therapeutic target and treatment strategy for ALI.

**Supplementary Information:**

The online version contains supplementary material available at 10.1186/s40779-025-00618-y.

## Background

The liver possesses a unique capacity for robust regeneration following acute liver injury (ALI). The rapid and efficient initiation of liver regeneration is crucial for recovery from ALI; otherwise, acute liver failure may occur [1, 2]. Liver regeneration encompasses distinct mechanisms that differ according to injury etiology [3]. Systemic metabolic remodeling and organ crosstalk induced by ALI are critical for liver regeneration [4–7]. One of the well-defined pathways involved in this process is the adipose-liver axis, in which lipolysis contributes to fuel, cell membrane synthesis substrates, and even transcriptional and epigenetic regulation to promote liver regeneration after ALI [8–10]. Moreover, the gut microbiota generates short-chain fatty acids [11], which are pivotal for hepatic membrane phospholipid biosynthesis during liver regeneration [12]. Of note, as one of the largest organs, skeletal muscle exhibited a response as drastic as adipose during ALI [9]. Importantly, low skeletal muscle mass has been shown to be an unfavorable factor for liver regeneration after liver transplantation [13, 14]. However, the detailed contribution of skeletal muscle and the muscle-liver axis remains elusive in ALI.

Glucocorticoids (GCs) are the most widely used anti-inflammatory drugs [15]. However, they have many side effects, such as osteoporosis, hypertension, and muscle atrophy [16–19]. GCs exert their functions by binding to the cytoplasmic GC receptor (GR) and then translocating to the nucleus as a transcription or co-transcription factor [20, 21]. Various studies in rodents have demonstrated the multifaceted effects of GCs on liver regeneration [22–24]. Importantly, GCs are commonly used to treat ALI, but their clinical outcomes remain controversial [25]. Given the extensive and complex functions of GCs, targeting the potential downstream molecules of GCs rather than the GCs themselves may be more efficient.

In this study, we focus on investigating the molecular mechanisms underlying liver repair and regeneration mediated by skeletal muscle-liver crosstalk during ALI, ultimately aiming to establish innovative therapeutic modalities and identify druggable targets for clinical translation.

## Methods

### Mice

Eight-week-old male C57BL/6J mice (*n* = 353) and 8–10 weeks old wild-type (WT) mice (*n* = 83), and *Fgf6* whole-body knockout (KO) mice (*n* = 94) (C57BL/6JGpt-Fgf6em3Cd1317/Gpt; Strain No. T015560) were purchased from GemPharmatech Co., Ltd. (Nanjing, China). The mice were maintained in a specific pathogen-free animal facility in a standard humidity- and temperature-controlled environment under a 12 h/12 h light/dark cycle, with free access to food and water. The partial (2/3) hepatectomy (PHx) model in rodents is a well-established system for studying liver regeneration, as it triggers a synchronized and reproducible proliferative response in hepatocytes [26]. In comparison, drug-induced ALI, including acetaminophen (APAP) overdose, initiates a compensatory regenerative response in the liver that serves as an adaptive mechanism, and the APAP-induced ALI model is particularly valuable for understanding regeneration in the context of pathological injury [27]. The combination of these two models enables a more comprehensive analysis of the regulatory mechanisms in the repair and regeneration processes following ALI. For the PHx model, two-thirds of the mouse liver was removed, as previously reported [28]. Briefly, the mice were subjected to gas anesthesia using 5% isoflurane for induction and 2% isoflurane for maintenance. A ventral midline incision was made to expose the abdominal cavity. The median and left lateral hepatic lobes were exteriorized. The mice were then ligated, and the incision wounds were sterilized with Betadine after closing the abdominal cavity on a warming pad set at 37 °C for recovery. The percentage change in tissue weight is calculated by subtracting the mean value of the control group from the mean value of the treatment group, then dividing the result by the mean value of the control group and multiplying by 100 to express it as a percentage. For the ALI model, mice were fasted overnight prior to APAP administration. The mice were then intraperitoneally injected with APAP (T0065; TargetMol, Shanghai, China) at a final dose of 300 mg/kg body weight and euthanized 24 or 48 h post-APAP administration. All animal experiments were performed according to the procedures approved by the Animal Care Committee of Shanghai Sixth People’s Hospital Affiliated to Shanghai Jiao Tong University (2023–0503).

### Human samples

Serum samples from patients (*n* = 33) with ALI were obtained from the Shanghai Sixth People’s Hospital Affiliated to Shanghai Jiao Tong University. For each ALI patient included in the study, we collected serum samples only on the first day to measure fibroblast growth factor binding protein 1 (FGFBP1) levels. The hepatic enzyme data were derived from clinical diagnostic records obtained during the patient’s hospitalization. Multiple time-point measurements of hepatic enzymes were used to calculate the slope of hepatic enzyme changes for each patient. This calculated slope was then correlated with the serum FGFBP1 levels measured on the first day. The characteristics of these patients were described in the Additional file 1: Table S1. The protocol was approved by the Human Ethics Committee of Shanghai Sixth People’s Hospital Affiliated to Shanghai Jiao Tong University [2024-KY-147(K)].

### Cell culture and treatment

To isolate primary mouse myoblasts, newborn (P0–P1) mice were euthanized, and their hind limb skeletal muscles were collected. The muscles were minced and digested in 0.2% collagenase type 2 solution (LS004176, Worthington Biochemical, NJ, USA) at 37 °C with shaking at 150 rpm for 30 min. The tissue suspension was then filtered and digested with 0.25% trypsin at 37 °C with shaking at 80 rpm for 20 min. The cells were further centrifuged and purified repeatedly from fibroblasts using the differential adhesion method. The isolated primary myoblasts were grown on Matrigel-coated plates in DMEM containing 20% FBS (10099, Gibco, NY, USA), 10% horse serum (E510006, Sangon Biotech, Shanghai, China), and 1% P/S (15140–122, Gibco, NY, USA) at 37 °C with 5% CO_2_. Differentiation was induced by replacing the medium of confluent myoblasts with DMEM containing 2% horse serum and 1% P/S for 48 h. For Dex administration, differentiated myoblasts were treated with Dex (D4902, Sigma, MO, USA) or vehicle (ethanol) as a control, and the cells were cultured at the indicated times. For the FGFBP1 release experiment, differentiated myoblasts were treated with or without heparin (5.5 μg/ml) and rFGF6 (1 or 2 μmol/L) for 2 h, and the culture medium and cells were collected for further analyses.

The C2C12 cells were grown in DMEM containing 10% FBS (10099, Gibco, NY, USA) and 1% P/S (15140–122, Gibco, NY, USA) at 37 °C with 5% CO_2_. Differentiation was induced by replacing the medium of confluent myoblasts with DMEM containing 2% heparan sulfate and 1% P/S for 48 h. The *Atf3* knockdown adenovirus [29] or ATF3 overexpression lentivirus was added at day 4 after differentiation. The rFGF6 (200 ng/ml) or MEK inhibitor (10 nmol/L) (MEKi; PD0325901, TargetMol, Shanghai, China) was treated at day 6 after differentiation for 12 h. Cells were collected for qRT-PCR and Western blotting analysis.

AML12 cells were maintained in DMEM/F-12 medium (10565018, Gibco, NY, USA) supplemented with 10% FBS, 1% P/S, 0.005 mg/ml insulin, 0.005 mg/ml transferrin, 5 ng/ml selenium [insulin–transferrin–sodium (ITS) selenite; I3146, Sigma, MO, USA], and 40 ng/ml Dex (D4902, Sigma, MO, USA). For the cell proliferation assay, AML12 cells were seeded into 96-well plates and transfected with 18 FGF expression vectors (200 ng per well) using Lipofectamine 3000 (Invitrogen, CA, USA). rFGFBP1 (10 ng/ml; 1413-FB, R&D Systems, MN, USA) or FGFBP1Ab (2 μg/ml; AF1413, R&D Systems, MN, USA) was then added to cells 24 h after transfection. Cell proliferation was detected using a BrdU Cell Proliferation Assay Kit (6813S, Cell Signaling Technology, MA, USA) according to the manufacturer’s instructions. AML12 cells were seeded into 96-, 48- and 24-well plates and replaced the medium with serum-free DMEM after 24 h, then treated with rFGFBP1 (10 ng/ml), rFGF5 (100 ng/ml), or rFGF5s (100 ng/ml) for 12 h. Cells were used for CCK-8 determination, Western blotting analysis of p-ERK, and immunofluorescence analysis of Ki67.

### HLO generation and functional studies

We used the 1016 human induced pluripotent stem cells (hiPSCs) clones (kindly provided by the Department of Stem Cell and Regenerative Biology at Harvard University and Harvard Stem Cell Institute) for HLO generation as previously described [30]. Briefly, for differentiation, hiPSCs were grown on Geltrex (A1413202, Gibco, NY, USA) coated tissue culture plates in RPMI 1640 (C11875500CP, Gibco, NY, USA) containing: day 1, 100 ng/ml activin A (120-14E, Peprotech, NJ, USA) and 50 ng/ml bone morphogenetic protein 4 (120–05, Peprotech, NJ, USA); day 2, 100 ng/ml activin A and 0.2% FBS (16000–044, Gibco, NY, USA); day 3, 100 ng/ml activin A and 2% FBS. On day 4 to 6, cells were cultured in advanced DMEM/F12 (12634028, Gibco, NY, USA) with 1× B27 (Gibco, 12587010), 1× N2 (17502048, Gibco, NY, USA), 1× GlutaMaX (35050–061, Gibco, NY, USA), 500 ng/ml FGF4 (AF-100–31, Peprotech, NJ, USA), 3 μmol/L CHIR99021 (SML1046, Sigma, MO, USA) and 1% P/S (C0222, Beyotime, Shanghai, China) and the medium was replaced every day. For the HLO induction, the foregut cells were collected and suspended in Matrigel (356237, Corning, MI, USA). A total of 100,000 cells were embedded in 50 μl Matrigel drop on the dishes in advanced DMEM/F12 with 1× B27, 1× N2, 1× GlutaMaX, 5 ng/ml FGF2 (100-18B, Peprotech, NJ, USA), 10 ng/ml VEGF (100–20–10, Peprotech, NJ, USA), 20 ng/ml EGF (AF-100–15, Peprotech, NJ, USA), 3 μmol/L CHIR99021, 0.5 μmol/L A83–01 (2939, Tocris, MO, USA), 50 μg/ml ascorbic acid (A5960, Sigma, MO, USA) and 1% P/S for 4 d. Then the media was switched to advanced DMEM/F12 with 1× B27, 1× N2, 1× GlutaMaX, 2 μmol/L retinoic acid (R2625, Sigma, MO, USA), and 1% P/S and cultured for another 4 d. Finally, organoids were harvested and re-embedded in Matrigel on the ultra-low attachment multiwall plate (3471, Corning, MI, USA) in liver maturation media [hepatocyte culture medium (CC-3198, Lonza, MD, USA) prepared as manufacturer’s instructions, except no EGF, and supplemented with 10 ng/ml hepatocyte growth factor (100–39, PeproTech, NJ, USA), 0.1 μmol/L Dex (D4902, Sigma, MO, USA), 20 ng/ml oncostatin M (300–10, Peprotech, NJ, USA), and 1% P/S] with PBS or rFGFBP1 (25 ng/ml) or rFGFBP1 (25 ng/ml) + rFGF5 (200 ng/ml) for 10 d. Cells were maintained at 37 °C in humidified air with 5% CO_2_, and the medium was added every 2 d.

For further details regarding the materials and methods used, please refer to Additional file 1: Methods.

### Statistical analysis

Statistical analyses were performed using GraphPad Prism 7.0 software. All data for comparative analysis of intergroup differences are presented as mean ± standard error of the mean (SEM), while the data of patient demographics and clinical characteristics uses mean ± standard deviation (SD) to directly reflect individual variability. The differences between the two groups were first assessed for normality using the Shapiro–Wilk test and for homogeneity of variance using Levene’s test. For data that did not meet these assumptions, log transformation was applied, after which all data satisfied the assumptions of normality and homogeneity of variance. Finally, an unpaired Student’s *t*-test was employed to statistically analyze the differences between the two groups. Two-way ANOVA followed by Bonferroni’s multiple comparison test was used for multiple group comparisons. Categorical variables were analyzed using a chi-square test. Correlations were examined using the nonparametric Spearman’s correlation test. A *P*-value < 0.05 was considered statistically significant.

## Results

### ALI induces GC-mediated transrepression of *Fgf6* in the skeletal muscle

**Fig. 1:**
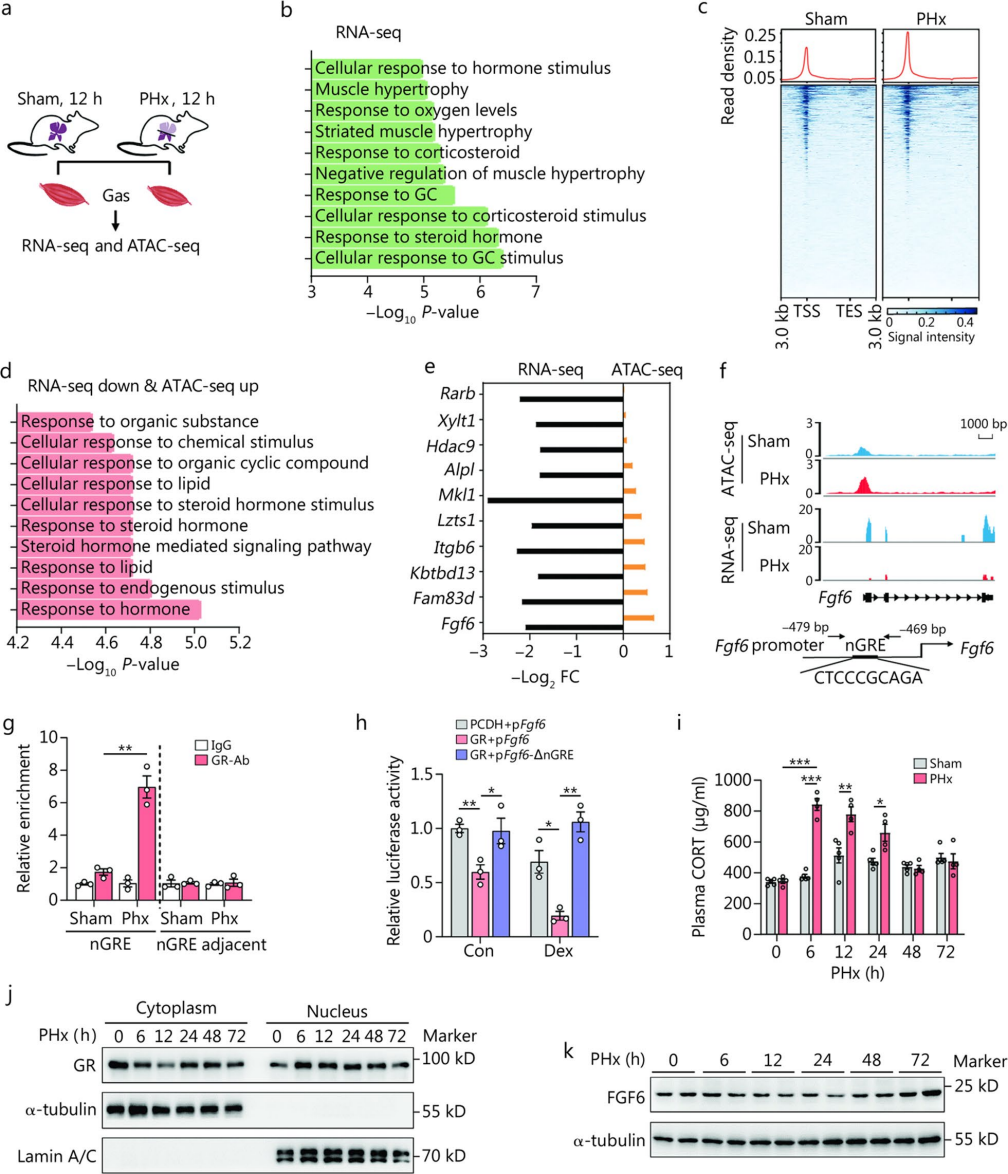
Acute liver injury (ALI) induces glucocorticoid (GC)-mediated transrepression of *Fgf6* in skeletal muscle. **a** Schematic of partial (2/3) hepatectomy (PHx) or sham surgery in 8-week-old male C57BL/6J mice, where the gastrocnemius (Gas) muscles were obtained for RNA-sequencing (RNA-seq) and assay for transposase-accessible chromatin by sequencing (ATAC-seq) analysis 12 h post-PHx. **b** Top 10 Gene Ontology (GO) enrichment terms of the differentially expressed genes (DEGs). **c** ATAC-seq signals in the Gas 12 h after PHx and sham surgery in mice. Genes shown in the rows were sorted in decreasing order by the signal intensity in each condition. **d** GO analysis of genes annotated to GC-related processes. ATAC-seq up indicates increased chromatin accessibility. **e** Top 10 genes with peaks (nucleosome-free regions) in the promoter region and decreased expression. **f** Visualization of ATAC-seq and RNA-seq data in the proximal region of the *Fgf6* in the Gas and the putative negative GC receptor (GR)-responsive element (nGRE) in the *Fgf6* promoter. **g** Chromatin immunoprecipitation (ChIP) assay assessing GR binding on the putative nGRE region of the *Fgf6* promoter in the Gas of C57BL/6 mice 12 h after PHx or sham surgery (*n* = 3). **h** GR activation induced wild-type *Fgf6* transcriptional activity, not an nGRE mutant reporter (*Fgf6*-ΔnGRE) (*n* = 3). **i** Plasma concentration of corticosterone (CORT) over time post-PHx (*n* = 5). **j** GR protein levels in the cytoplasm and nucleus of Gas at the indicated times after PHx (*n* = 3 independent biological replicates). **k** FGF6 protein levels and their corresponding quantification in the Gas at the indicated times after PHx (*n* = 2). ^*^P < 0.05, ^**^P < 0.01, ^***^P < 0.001. TSS transcription start site, TES transcription end site, FGF6 fibroblast growth factor 6, GR-Ab glucocorticoid receptor antibody, PCDH plasmid cloning and delivery vector with hygromycin, p*Fgf6 Fgf6* promoter, FC fold change

A rapid metabolic response to ALI induces the loss of fat and lean masses to their nadirs at 24 h after PHx [9]. In this study, we first examined the liver and muscle parameters of mice at 12 h after PHx or sham surgery and confirmed that muscle weight [gastrocnemius (Gas), 11%; tibia anterior (Ta), 11%; quadriceps (Qu), 10%; soleus (Sol), 17%], as well as protein synthesis rate (Gas, 49%; Ta, 30%; Qu, 47%) were decreased, but the liver protein synthesis rate (92%) significantly increased (Additional file 1: Fig. S1). To determine the main cause of muscle loss during ALI, we performed RNA-sequencing (RNA-seq) and assay for transposase-accessible chromatin by sequencing (ATAC-seq) analyses of the mouse Gas 12 h after PHx or sham surgery (Fig. 1a). We identified a total of 1016 significantly differentially expressed genes (DEGs) based on a false discovery rate (FDR) < 0.05 and |log_2_ fold change (FC)|> 1. Among these, 616 were up-regulated and 400 were down-regulated in the PHx group compared to the sham group (Additional file 2). Intriguingly, Gene Ontology (GO) analysis revealed that GC-mediated regulation plays a dominant role in the muscles after PHx and that the chromatin accessibility of the PHx group was more open than the sham group (Fig. 1b, c). Further combined analysis of the RNA-seq and ATAC-seq data showed that these genes exhibiting concurrent downregulation of transcriptional activity and enhanced chromatin accessibility demonstrated significant enrichment in GC-associated regulatory pathways (Fig. 1d). Therefore, we focused on these genes that have decreased expression and nucleosome-free regions (“peaks”) in their promoter regions, revealing *Fgf6* as the top enriched gene (Fig. 1e; Additional file 2).

A recent study reported the widespread direct transrepression function of GCs through binding to negative GC response elements (nGREs) [31]. We identified a putative nGRE based on the accessible peaks in the promoter of *Fgf6* (Fig. 1f). Subsequent chromatin immunoprecipitation (ChIP) assays confirmed specific GR binding to the putative nGRE region of the *Fgf6* promoter without activating the adjacent region. Importantly, GR binding was significantly enhanced by PHx treatment (Fig. 1g). As shown in the results of the luciferase assay, GR overexpression significantly repressed *Fgf6* transcriptional activation, whereas mutations in the putative nGRE abolished transrepression (Fig. 1h). Furthermore, in vitro and in vivo experiments using dexamethasone (Dex) also showed repressed *Fgf6* expression (Additional file 1: Fig. S2a, b).

We further investigated the circulatory concentration of GCs and the nuclear translocation of the GR in the muscle during ALI. As shown in Fig. 1i, j and Additional file 1: Fig. S2c–e, plasma corticosterone (CORT) levels were highest at 6 and 12 h in mice subjected to PHx and APAP-induced liver injury (AILI), respectively, approximately 3-time higher than their basal levels, concomitant with an elevation in nuclear GR protein abundance. Correspondingly, the protein levels of FGF6 showed a dynamic decline and recovery process (Fig. 1k; Additional file 1: Fig. S2f, g). These results indicate that GCs may dominate the metabolic alteration of muscles via the transrepression of *Fgf6* during ALI in mice.

### Activation of GR signaling in muscle after ALI induces muscle loss and is beneficial to ALI repair

**Fig. 2:**
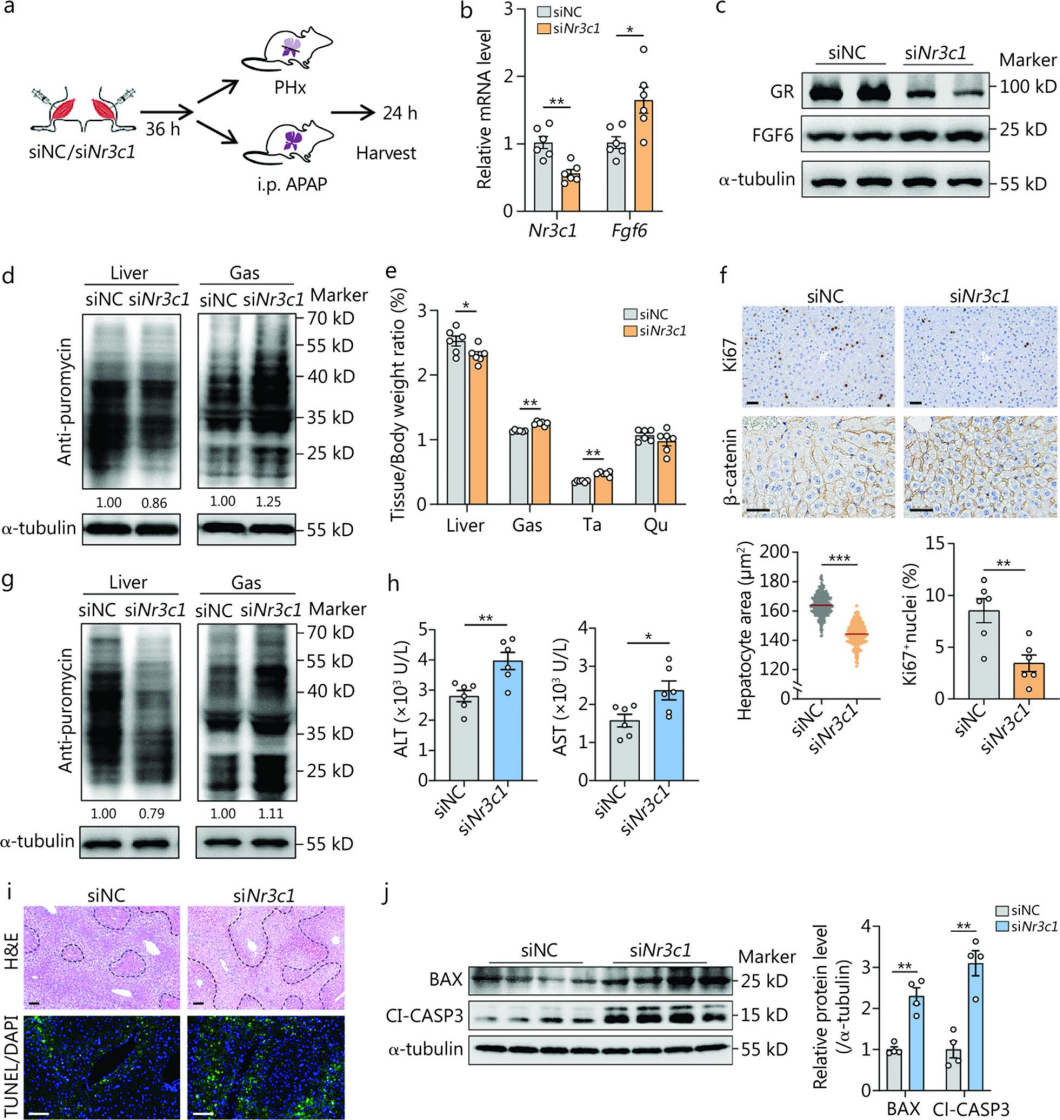
Skeletal muscle glucocorticoid receptor (*GR*) knockdown via siRNA delivery exacerbates acute liver injury (ALI) in mice. **a** Schematic of the animal experiments. Briefly, 8-week-old C57BL/6 mice were injected with si*Nr3c1* (*Nr3c1*, encoding GR) or siNC into the gastrocnemius (Gas) and tibialis anterior (Ta) muscles and subjected to partial (2/3) hepatectomy (PHx) or acetaminophen (APAP). **b** mRNA levels of *Nr3c1* and *Fgf6* in the Gas of mice 24 h after PHx (*n* = 6). **c** Protein levels of GR and FGF6 in the Gas of mice 24 h after PHx (*n* = 2). **d** Western blotting analysis depicting the changes in protein synthesis rates [tracked using the surface sensing of translation (SUnSET) assay] in the liver and Gas of mice 24 h after PHx (*n* = 3 independent biological replicates). The levels of puromycin relative to the α-tubulin were shown through quantification, with the level of puromycin in the siNC setting as 1.00. This applied to all puromycin quantification in this figure. **e** Tissue weight as a percentage of body weight in the liver, Gas, Ta, and quadriceps (Qu) of mice 24 h after PHx (*n* = 6). **f** Representative Ki67 (for indicating the proliferating cells) and β-catenin (for indicating cell size) immunohistochemistry of mouse liver Sections 24 h after PHx, and quantification of hepatocyte area and Ki67^+^ nuclei (*n* = 6). Scale bar = 50 μm.** g** Western blotting analysis of the protein synthesis rates in the liver and Gas of mice 24 h after APAP dosing (*n* = 3 independent biological replicates). **h** Serum concentrations of alanine aminotransferase (ALT) and aspartate aminotransferase (AST) (*n* = 6). **i** Representative hematoxylin and eosin (H&E) (necrotic areas circled with black lines) and terminal deoxynucleotidyl transferase dUTP nick-end labeling (TUNEL) staining (for identifying apoptotic cells) images of mouse liver Sections 24 h after APAP dosing. Scale bar = 50 μm. **j** Western blotting analysis of hepatic apoptosis related proteins BCL2 associated X (BAX) and cleaved caspase-3 (Cl-CASP3) expression with the corresponding quantification (*n* = 4). ^*^P < 0.05, ^**^P < 0.01, ^***^P < 0.001. FGF6 fibroblast growth factor 6, Nr3c1 nuclear receptor subfamily 3 group c member 1, DAPI 4’,6-diamidino-2-phenylindole

Next, we investigated whether activated GR signaling in muscles plays an important role in ALI repair. First, we delivered a specific siRNA targeting *Nr3c1* (si*Nr3c1*, encoding GR) into the Gas and Ta of the mice to investigate if it is essential in muscular metabolic alteration upon ALI (Fig. 2a). Compared to treatment with scramble controls (siNC), intramuscular injection of *Nr3c1*-knockdown siRNA significantly reduced GR expression in skeletal muscle, while having no effect on hepatic GR expression (Fig. 2b, c**; **Additional file 1: Fig. S3). This intervention differentially modulated protein synthesis rates in liver and muscle tissues, with a 14% decrease in liver and a 25% increase in skeletal muscle (Fig. 2d). Importantly, the Liver/Body weight ratio (9%) was significantly reduced in PHx mice with GR deficiency, whereas the Gas/Body weight (10%) and Ta/Body weight (34%) ratios were increased (Fig. 2e). Hepatocyte hypertrophy and proliferation are the key events in the process of liver regeneration [32, 33]. Indeed, GR deficiency in Gas and Ta impaired liver regeneration in mice, as shown by the decreased hepatocyte area and few Ki67^+^ nuclei (Fig. 2f). Consistently, GR deficiency in the Gas and Ta exacerbated AILI compared to siNC treatment, as shown by a 21% decrease in hepatic protein synthesis and an 11% increase in skeletal muscle protein synthesis, elevated serum ALT and AST levels, and more severe apoptosis (Fig. 2g–j). Furthermore, we used nandrolone phenylpropionate (steroids, promotes protein synthesis), and mifepristone (GR antagonist) to further determine the muscle-liver crosstalk during liver regeneration. As shown in Additional file 1: Fig. S4, nandrolone or mifepristone administration in skeletal muscle resisted the PHx-induced muscle loss and impaired liver regeneration. We then intramuscularly administered low-dose Dex in mice to investigate its effect on ALI but found no significant effect in either PHx or AILI mice (Additional file 1: Fig. S5). These results reveal the critical role of GCs in mediating metabolic alterations in the muscles after ALI and that GCs are essential for liver regeneration. However, exogenous glucocorticoid supplementation may confer no additional benefit when endogenous glucocorticoid levels are already elevated, though it could still be beneficial in individuals with impaired endogenous glucocorticoid release.

### FGF6 acts downstream of GCs to mediate the muscle metabolic response to ALI and influence liver regeneration

FGF6 is predominantly expressed in skeletal muscles and plays key roles in myogenesis in an autocrine and/or paracrine manner [34, 35]. Our previous study suggested that FGF6 is an anabolic factor that promotes protein synthesis [36]. Therefore, in this study, we tested the effects of FGF6 on ALI in mice. First, we adapted an adenovirus-associated virus (AAV) system to specifically express a tissue-specific double muscle creatine kinase (*Mck*) promoter [37]-driven FGF6 expression in a plasmid in the Gas and Ta of mice. After 3 weeks, the mice were subjected to PHx, and the liver and Gas were collected at different time points after surgery (Additional file 1: Fig. S6a, b). Notably, FGF6 overexpression in the skeletal muscle decelerated liver regeneration after PHx, as demonstrated by a decrease in Liver/Body weight ratio (24 h, 10%; 48 h, 9%; 72 h, 5%) and hepatocyte proliferation, and an increase in the Gas/Body weight ratio at 24 h (7%) (Additional file 1: Fig. S6c–e). Consistently, recombinant FGF6 protein (rFGF6) delivery into the skeletal muscle after PHx at the indicated times also impaired liver regeneration (Additional file 1: Fig. S6f–i).

**Fig. 3:**
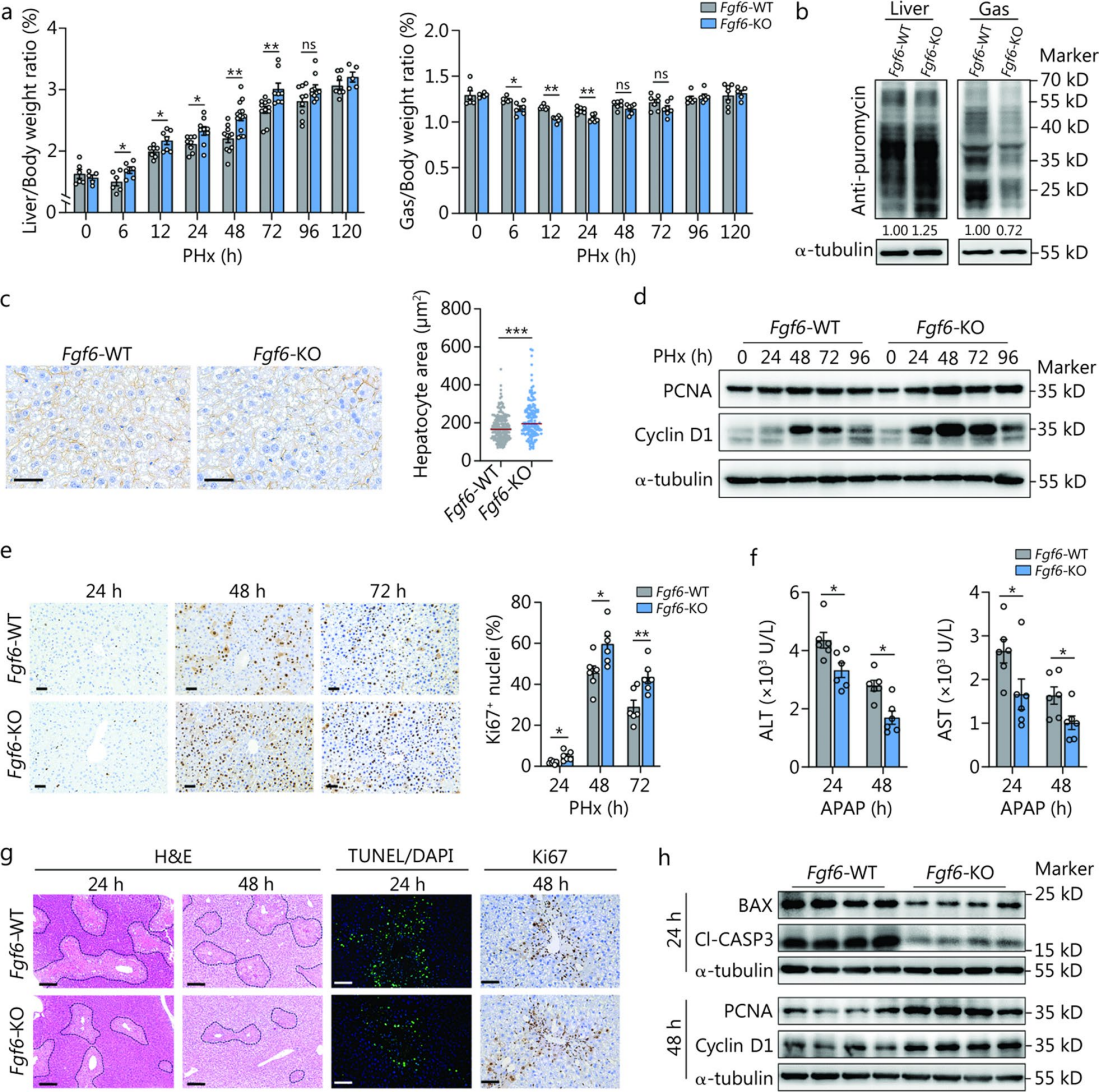
*Fgf6* deficiency protects mice from PHx- and APAP-induced acute liver injury (ALI). **a** Liver/Body weight ratio determined at the indicated time points (*n* = 5–14 per time point and group), and Gas/Body weight ratio as determined at the indicated time points (*n* = 5–7 per time point and group). **b** Western blotting analysis of the protein synthesis rates in the liver and Gas of WT and *Fgf6*- KO mice 24 h after partial (2/3) hepatectomy (PHx) (*n* = 3 independent biological replicates). The levels of puromycin relative to the α-tubulin were shown through quantification, with the level of puromycin in the WT setting as 1.00. **c** Representative liver β-catenin (for indicating cell size) immunohistochemistry results and quantification of the hepatocyte area (*n* = 5). Scale bar = 50 μm. **d** Western blotting analysis of the hepatic proliferation-related proteins PCNA and cyclin D1 (G1/S-specific) at the indicated time points after PHx (*n* = 3 independent biological replicates). **e** Representative liver Ki67 (for indicating the proliferating cells) immunohistochemistry results and quantification of Ki67^+^ nuclei (*n* = 6). Scale bar = 50 μm. **f** Serum concentrations of alanine aminotransferase (ALT) and aspartate aminotransferase (AST) in WT and *Fgf6*-KO mice 24 and 48 h after APAP dosing (300 mg/kg body weight; *n* = 6). **g** Representative liver H&E (necrotic areas circled with black lines), TUNEL (for identifying apoptotic cells), and Ki67 staining results 24 and 48 h after APAP dosing. Scale bars = 100 μm (H&E) or 50 μm (TUNEL and Ki67). **h** Western blotting analysis of hepatic apoptosis and proliferation-related proteins BAX, Cl-CASP3, PCNA, and cyclin D1 expression (*n* = 4). The data (**f**) were log-transformed and then analyzed using an unpaired Student’s *t*-test. ^*^P < 0.05, ^**^P < 0.01, ^***^P < 0.001, ns non-significant. Fgf6 fibroblast growth factor 6, APAP acetaminophen, PCNA proliferating cell nuclear antigen, H&E hematoxylin and eosin, TUNEL terminal deoxynucleotidyl transferase dUTP Nick-End labeling, BAX BCL2 associated X, Cl-CASP3 cleaved caspase-3, FGF6 fibroblast growth factor 6, Gas gastrocnemius, WT wild-type, KO knockout, DAPI 4’,6-diamidino-2-phenylindole

Next, we used WT and *Fgf6*-KO mice subjected to PHx or AILI conditions to further elucidate the role of FGF6 in mediating liver regeneration in ALI. We and others have shown that *Fgf6*-WT and *Fgf6*-KO mice do not exhibit metabolic abnormalities under normal conditions [38, 39]. Notably, we found that *Fgf6* deficiency increased the Liver/Body weight ratio as early as 6 h after PHx and lasted until 72 h after surgery (Fig. 3a). Correspondingly, there was a significant decrease in the Gas/Body weight ratio from 6 to 24 h, as well as in the Ta and Qu at 24 h after PHx (Fig. 3a; Additional file 1: Fig. S7a). Detailed analysis revealed increased liver protein synthesis and suppression of protein synthesis in the Gas, which resulted in hepatocyte hypertrophy in *Fgf6*-KO mice compared to *Fgf6-*WT mice (Fig. 3b, c**; **Additional file 1: Fig. S7b, c). Importantly, *Fgf6* deficiency resulted in sustained hepatocyte proliferation in the liver after PHx (Fig. 3d, e**; **Additional file 1: Fig. S7d). Furthermore, we subjected *Fgf6*-WT and -KO mice to AILI. The results revealed that *Fgf6*-KO mice had alleviated ALI compared to WT mice, as shown by reduced serum concentrations of ALT and AST, damaged areas, and apoptosis, as well as increased cell proliferation (Fig. 3f–h**; **Additional file 1: Fig. S7e).

**Fig. 4:**
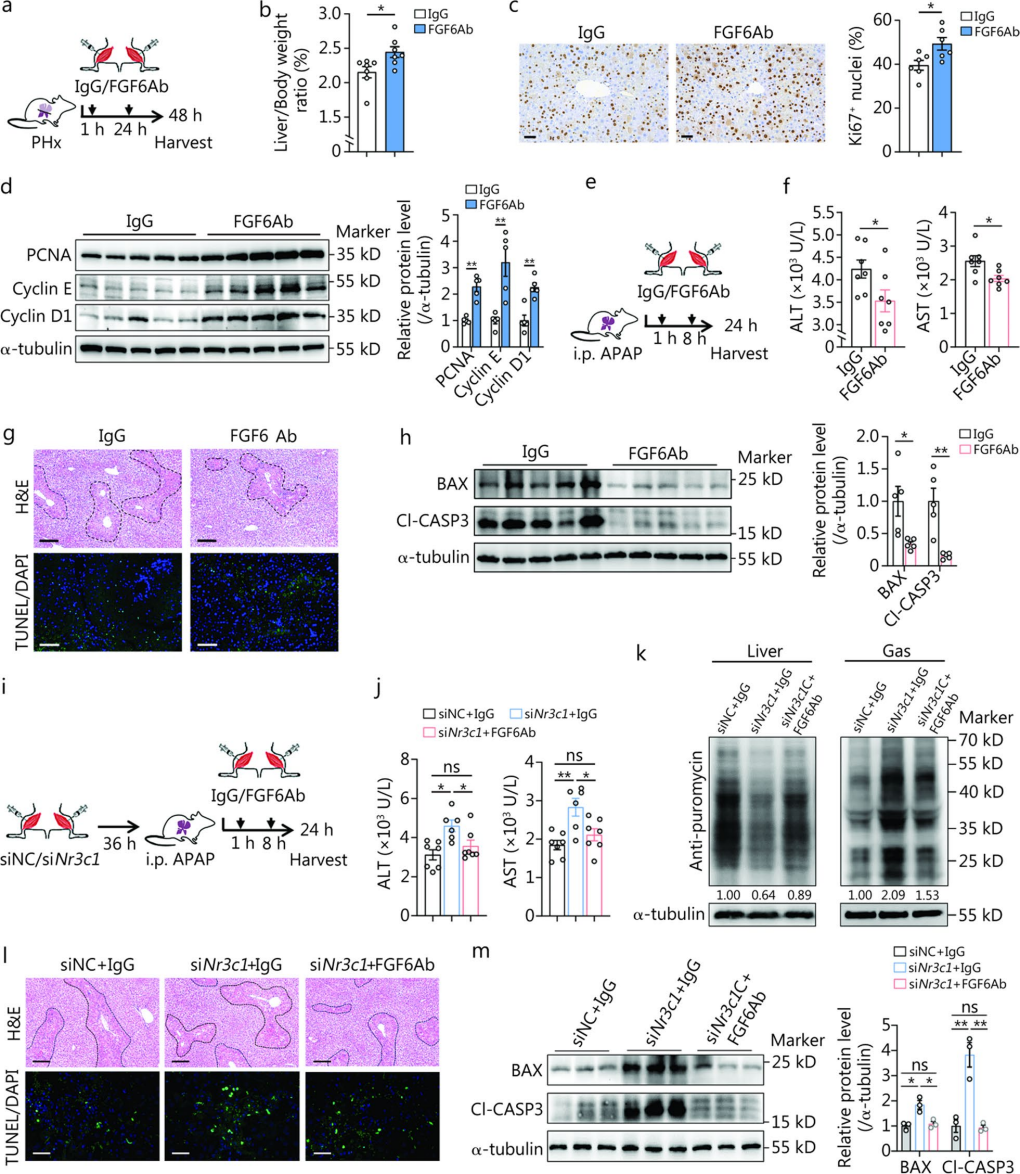
Fibroblast growth factor 6 (FGF6) acts downstream of GCs to mediate the muscle metabolic response to acute liver injury (ALI) and influence liver regeneration. **a** Schematic of FGF6-neutralizing antibody (FGF6Ab) or IgG (2 mg/kg body weight) administration in the Gas and Ta depots of partial (2/3) hepatectomy (PHx) mice. **b** Liver/Body weight ratio in the IgG and FGF6Ab treated mice at the indicated time points after PHx (*n* = 6). **c** Representative liver Ki67 (for indicating the proliferating cells) immunohistochemistry results and quantification of Ki67^+^ nuclei (*n* = 6). Scale bar = 50 μm. **d** Western blotting analysis of hepatic proliferation-related proteins PCNA, cyclin E, and cyclin D1 expression with the corresponding quantification (*n* = 5). **e** Schematic of FGF6Ab or IgG (2 mg/kg body weight) administration in the Gas and Ta muscles of acetaminophen (APAP)-treated mice. **f** Serum concentrations of alanine aminotransferase (ALT) and aspartate aminotransferase (AST) (*n* = 7). **g** Representative liver H&E (necrotic areas circled with black lines) and TUNEL (for identifying apoptotic cells) staining results 24 h after APAP dosing. Scale bars = 100 μm (H&E) or 50 μm (TUNEL). **h** Western blotting analysis of hepatic apoptosis-related proteins BAX and Cl-CASP3 expression with the corresponding quantification (*n* = 5). **i** Schematic of the animal experiments. si*Nr3c1* or siNC was delivered into the Gas and Ta depots of mice, subjected to APAP treatment, and then treated with FGF6Ab or IgG (2 mg/kg body weight) at the indicated times. **j** Serum concentrations of ALT and AST (*n* = 7). **k** Western blotting analysis of the protein synthesis rates in the liver and Gas (*n* = 3 independent biological replicates). The levels of puromycin relative to the α-tubulin were shown through quantification, with the level of puromycin in the siNC + IgG setting as 1.00. **l** Representative liver H&E (necrotic areas circled with black lines) and TUNEL staining results 24 h after APAP dosing. Scale bars = 100 μm (H&E) or 25 μm (TUNEL). **m** Western blotting analysis of hepatic apoptosis-related proteins BAX and Cl-CASP3 expression with the corresponding quantification (*n* = 3). The data (**d, h**) were log-transformed and then analyzed using an unpaired Student’s *t*-test. ^*^P < 0.05, ^**^P < 0.01, ns non-significant. GCs glucocorticoids, PCNA proliferating cell nuclear antigen, H&E hematoxylin and eosin, TUNEL terminal deoxynucleotidyl transferase dUTP Nick-End labeling, BAX BCL2 associated X, Cl-CASP3 cleaved caspase-3, Gas gastrocnemius, Ta tibialis anterior, DAPI 4’,6-diamidino-2-phenylindole

Finally, we tested whether the therapeutic inhibition of FGF6 was effective in improving ALI by administering FGF6-neutralizing antibody (FGF6Ab) to the mouse Gas and Ta muscles. When subjected to PHx, mice treated with FGF6Ab showed a significant increase in the Liver/Body weight ratio (13%), the proportion of Ki67^+^ nuclei, and cell cycle-related protein [proliferating cell nuclear antigen (PCNA), cyclin E, and cyclin D1] levels compared to mice treated with isotype control IgG (Fig. 4a–d). Consistently, FGF6Ab-treated AILI mice exhibited lower serum concentrations of ALT and AST, as well as decreased damaged areas, cell apoptosis, and BAX and cleaved caspase-3 (Cl-CASP3) protein levels in the liver compared to those in the control (Fig. 4e–h).

As a downstream target of GCs, we aimed to unravel the role of FGF6 in mediating the metabolic functions of GCs in the muscle during AILI. To achieve this, we delivered si*Nr3c1* or siNC in the Gas and Ta, subjected the mice to AILI for 36 h after siRNA delivery, and treated them with FGF6Ab or IgG (Fig. 4i). Notably, FGF6Ab treatment in the Gas and Ta of GR-deficient mice rescued the adverse effects of AILI, as compared to control mice treated with IgG (Fig. 4j–m). Taken together, these results emphasize the importance of GC signaling in the skeletal muscle against ALI via the transrepression of FGF6.

### FGFBP1 is the downstream target of FGF6 in skeletal muscle after ALI

**Fig. 5:**
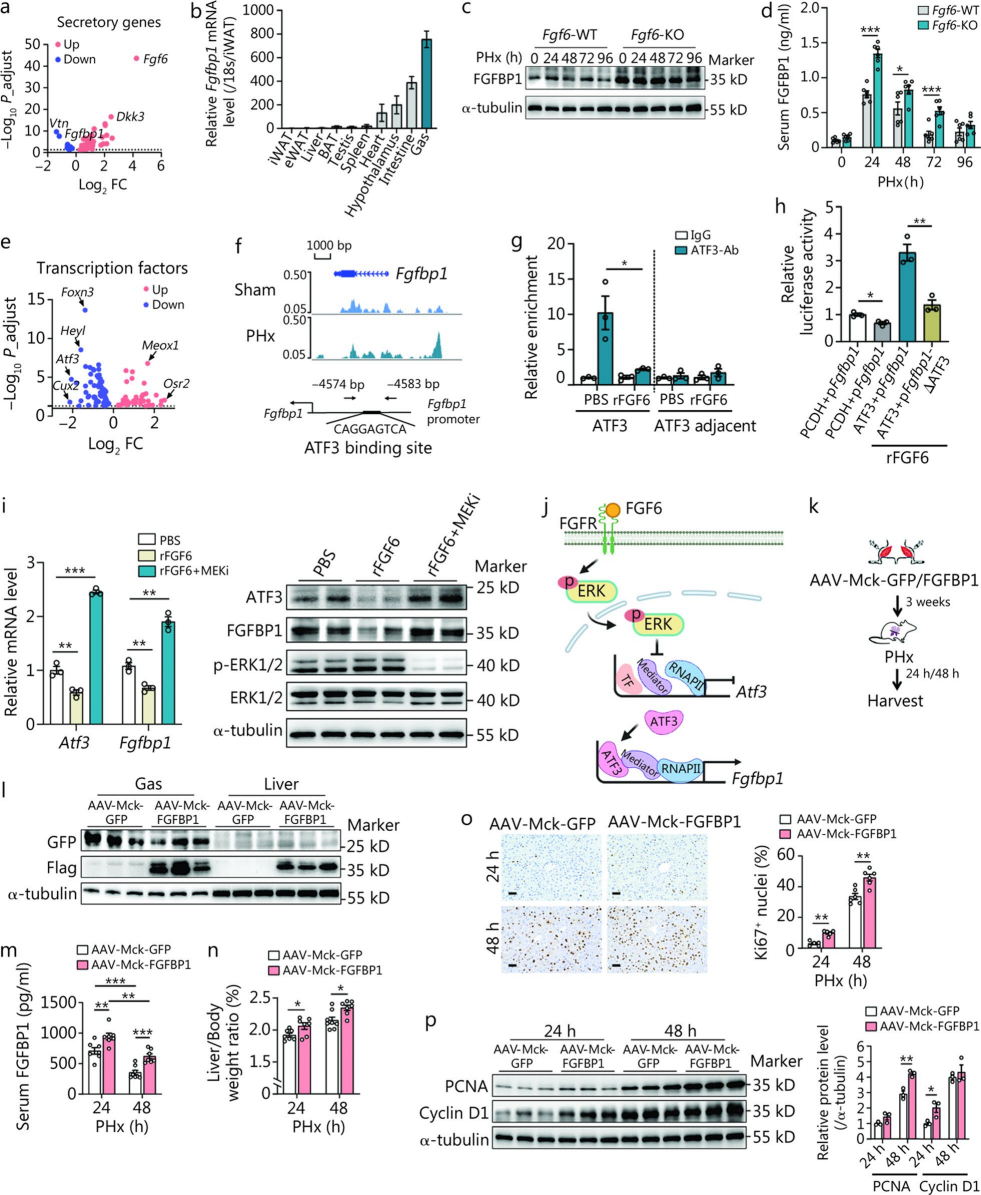
Fibroblast growth factor 6 (FGF6) controls the transcription of *Fgfbp1* via the ERK-ATF3 axis and skeletal muscle-derived FGFBP1 promotes liver regeneration.** a** Volcano plot showing the differentially expressed secretory genes identified in the Gas of the GFP- and FGF6-overexpression groups. The thresholds were set at |log_2_ fold change (FC)|> 0.2 and *P*_adjust < 0.05. **b** The mRNA expression pattern of *Fgfbp1* (*n* = 5). **c** Protein level of FGFBP1 in the Gas of *Fgf6*-WT and -KO mice at the indicated times after partial (2/3) hepatectomy (PHx) (*n* = 3 independent biological replicates). **d** Serum concentrations of FGFBP1 in the *Fgf6*-WT and -KO mice at the indicated time points after PHx (*n* = 6). **e** Volcano plot showing the differentially expressed transcription factors identified in the Gas of the GFP- and FGF6-overexpression groups. The thresholds were set at |log_2_ FC|> 0.2 and *P*_adjust < 0.05. **f** Visualization of ATAC-seq data in the proximal region of the *Fgfbp1* in the Gas 12 h after PHx or sham surgery, and the putative activating transcription factor 3 (ATF3)-responsive element in the *Fgfbp1* promoter within the peak region. **g** Chromatin immunoprecipitation (ChIP) assay assessing ATF3 binding on the putative region of the *Fgfbp1* promoter in the Gas of C57BL/6 mice 12 h after rFGF6 or PBS local injection (*n* = 3). **h** ATF3 activation rescued the inhibitory effect of rFGF6 on WT *Fgfbp1* transcriptional activity, but not an *Atf3* mutant reporter (*Fgfbp1*-ΔATF3) (*n* = 3). **i** Inhibition of extracellular signal regulated kinases (ERK) signaling abolished the inhibitory effect of rFGF6 on ATF3 and FGFBP1 expression (*n* = 3). **j** Proposed model for FGF6 downstream regulation of FGFBP1 via ERK-ATF3 cascade in muscle. Image created with BioRender.com. **k** Schematic of the animal experiments. AAV-Mck-FGFBP1 or AAV-Mck-GFP were administered to the Gas and Ta depots of 8-week-old C57BL/6 mice. PHx was performed 3 weeks later, and the livers and Gas were analyzed 24 and 48 h post-PHx. **l** Western blotting analysis of the expression of GFP and Flag in the Gas and liver (*n* = 3). **m** Serum concentration of FGFBP1 in the AAV-Mck-FGFBP1 and AAV-Mck-GFP mice at the indicated time points after PHx (*n* = 7). **n** Liver/Body weight ratio in the AAV-Mck-FGFBP1 and AAV-Mck-GFP mice at the indicated time points after PHx (*n* = 7). **o** Representative liver Ki67 immunohistochemistry results and quantification of Ki67^+^ nuclei (*n* = 6). Scale bar = 50 μm. **p** Western blotting analysis of hepatic proliferation-related proteins PCNA and cyclin D1 expression at the indicated time points after PHx with the corresponding quantification (*n* = 3). ^*^P < 0.05, ^**^P < 0.01, ^***^P < 0.001. GCs glucocorticoids, FGFBP1 fibroblast growth factor binding protein 1, PCNA proliferating cell nuclear antigen, rFGF6 recombinant FGF6 protein, Gas gastrocnemius, GFP green fluorescent protein, WT wild-type, KO knockout, ATAC-seq assay for transposase-accessible chromatin by sequencing, rFGF6 recombinant FGF6 protein, PBS phosphate-buffered saline, AAV adenovirus-associated virus, FGFR fibroblast growth factor receptor, RNAP II RNA polymerase II, TF transcriptional factor

Considering the sustained acceleration of liver regeneration in *Fgf6*-deficient mice after PHx, we hypothesized that a secretory factor in skeletal muscle mediates this effect. To achieve this, we analyzed the RNA-seq data of muscle with FGF6 overexpression and screened out all differential secretory genes (Fig. 5a**; **Additional file 1: Fig. S8a; Additional file 3). The expression of 3 top secretory genes, *Fgfbp1*, *Vtn*, and *Dkk3* was verified using qRT-PCR (Additional file 1: Fig. S8b). Further tissue expression pattern analysis showed that *Fgfbp1* was highly expressed in the skeletal muscle, *Vtn* was mainly expressed in the liver, and *Dkk3* was universally expressed (Fig. 5b**; **Additional file 1: Fig. S8c). Comprehensive characterization of DKK3 expression upon PHx showed that the DKK3 level decreased in the skeletal muscle and increased in the serum after PHx, while *Fgf6* deficiency did not affect the serum concentration of DKK3 (Additional file 1: Fig. S8d–f). In contrast, FGFBP1 levels showed a stereotypical pattern of increase and recovery in serum after PHx and AILI, whereas no change in FGFBP1 level was found in the intestine (Additional file 1: Fig. S8g–i). Notably, *Fgf6* deficiency significantly increased FGFBP1 protein levels in both the skeletal muscle and serum compared to WT mice after PHx (Fig. 5c, d**; **Additional file 1: Fig. S8j). These data indicated a repressive regulation of *Fgfbp1* by FGF6 in skeletal muscle.

To further elucidate the mechanism of FGF6 in regulating *Fgfbp1* transcription, we analyzed the RNA-seq data of muscle with FGF6 overexpression and focused on the top transcription factors (Fig. 5e**; **Additional file 3). Further ATAC-seq analysis of the transcriptional landscape of *Fgfbp1* promoter in the Gas 12 h after PHx or sham surgery showed an enriched peak, and in silico prediction revealed a putative activating transcription factor 3 (ATF3)-responsive element within the peak region (Fig. 5f). Subsequent luciferase and ChIP assays confirmed the activation of ATF3 to *Fgfbp1* transcription, while these effects were inhibited by rFGF6 (Fig. 5g, h). Echoing the results of in vitro knockdown of *Atf3* in C2C12 cells that resulted in decreased FGFBP1 expression (Additional file 1: Fig. S9a), while ATF3 overexpression abrogated the inhibitory effect of rFGF6 on FGFBP1 (Additional file 1: Fig. S9b). A recent study has revealed the direct transcriptional regulation of genes by extracellular signal regulated kinase (ERK), including ATF3 [40]. As a previous study [36] and our results showed a strong activation of ERK by rFGF6 in myogenic cells (Additional file 1: Fig. S9b), we confirmed this effect by using the MEK inhibitor in C2C12 cells (Fig. 5i; Additional file 1: Fig. S9c). Collectively, these results suggest that rFGF6 controls *Fgfbp1* transcription in the skeletal muscle via the ERK-ATF3 axis (Fig. 5j) and that FGFBP1 might be vital for liver regeneration upon ALI.

### Skeletal muscle-derived FGFBP1 was responsible for the *Fgf6* deficiency-mediated promotion of liver regeneration

To further test this hypothesis, we established a muscle-specific FGFBP1 overexpression model by administering an AAV with an *Mck* promoter expressing FGFBP1 with a fusion flag tag at the C-terminus and a non-fusion green fluorescent protein (GFP) (AAV-Mck-FGFBP1) or a control vector carrying GFP without a flag tag (AAV-Mck-GFP) into the Gas and Ta of mice (Fig. 5k). Three weeks after AAV injection, no differences in the body, liver, and Gas weights were observed (Additional file 1: Fig. S10a, b). Interestingly, AAV-Mck-FGFBP1 delivery achieved specific FGFBP1 overexpression in the Gas, as evidenced by the flag being detectable in the liver, but not GFP (Fig. 5l; Additional file 1: Fig. S10c). This result indicates that FGFBP1 was secreted from the skeletal muscle and moved to the liver. Mice overexpressing FGFBP1 showed significantly elevated FGFBP1 levels in the serum at 24 and 48 h after PHx (Fig. 5m), along with enhanced liver regeneration without affecting Gas weight (Fig. 5n–p**; **Additional file 1: Fig. S10d). We then treated *Fgf6*-KO mice with FGFBP1-neutralizing antibody (FGFBP1Ab) or isotype control IgG (KO + FGFBP1Ab and KO + IgG, respectively) and compared them with WT mice treated with IgG (WT + IgG) (Additional file 1: Fig. S11a). Following PHx, FGFBP1Ab administration to *Fgf6*-KO mice largely abolished the beneficial effects of *Fgf6* deficiency, promoting liver regeneration (Additional file 1: Fig. S11b–d). Overall, these results uncovered a previously unappreciated role of FGFBP1 in liver regeneration and the indispensable role of the FGF6-ATF3-FGFBP1 transcription axis in mediating muscle-liver crosstalk during ALI.

### FGFBP1 forms liquid-like condensates

FGFBP1 is a secreted protein that functions as a chaperone for FGF ligands from the extracellular matrix and enhances FGF activity at low ligand concentrations [41]. Interestingly, FGFBP1 is noncovalently bound to the cell membrane, as heparin treatment increases its release [42]. We noticed that during ALI, *Fgf6* deficiency promoted sustained expression of FGFBP1 in skeletal muscle (Fig. 5c), while serum FGFBP1 levels exhibited an initial increase followed by a subsequent decrease (Fig. 5d). In consistent, the AAV stably mediated the overexpression of FGFBP1, while the level of FGFBP1 in the serum showed a significant decrease at 48 h compared with 24 h (Fig. 5m). These findings prompted us to investigate whether FGFBP1 undergoes phase separation, affecting its release.

**Fig. 6:**
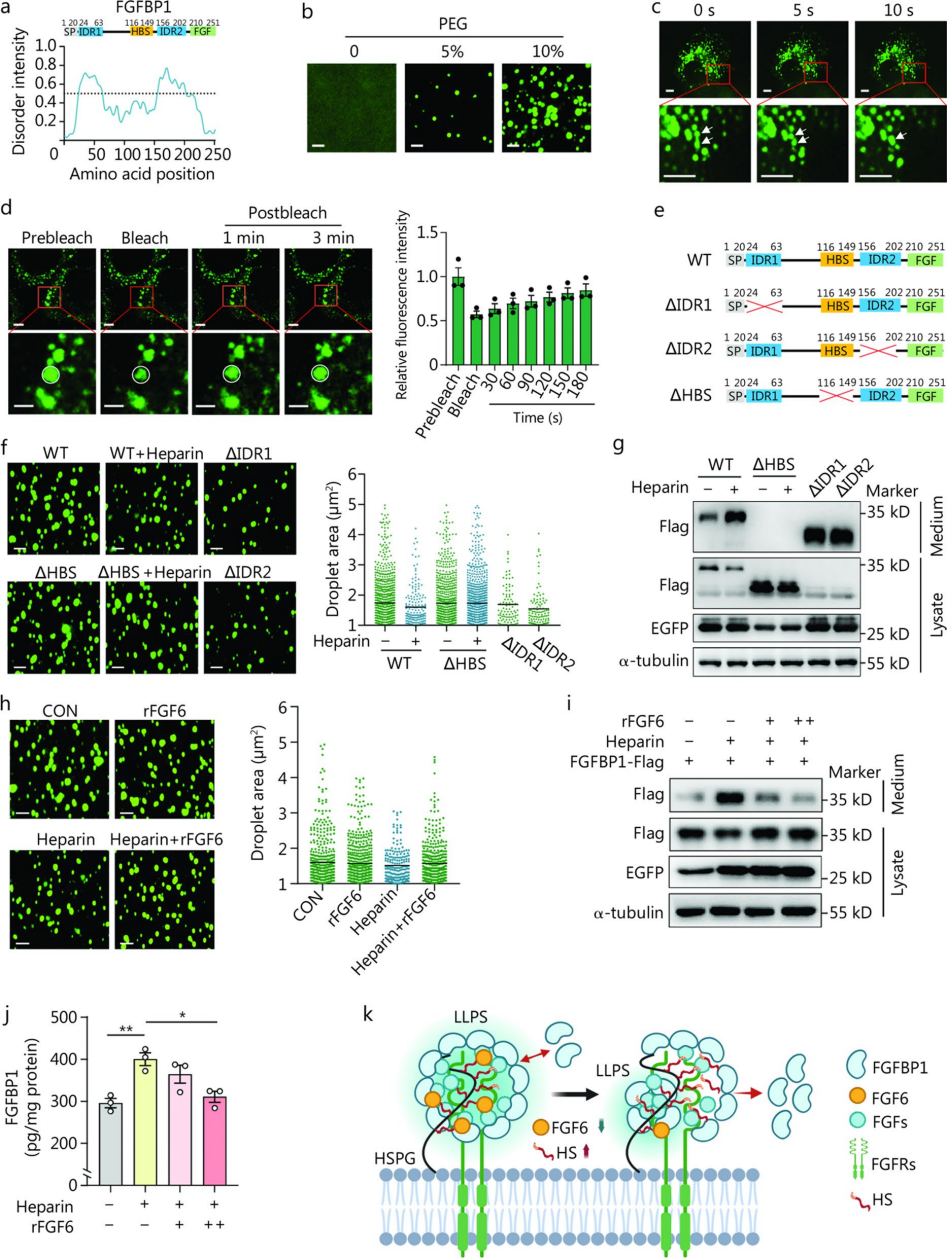
FGFBP1 undergoes liquid–liquid phase separation (LLPS) and is interfered with heparin. **a** The domain structure and the intrinsically disordered tendency of mouse FGFBP1 (lower). IUPred (https://iupred.elte.hu/) assigned scores of the disordered tendencies of the sequences between 0 and 1, with scores higher than 0.5 indicating disorder. The amino acid sequence of FGFBP1 contains two intrinsically disordered regions (IDRs). **b** Confocal microscopy images of the assembly status of 2 μmol/L purified rFGFBP1-EGFP with 0–5–10% PEG. Scale bar = 5 µm. **c** Representative images of the fusion of FGFBP1-EGFP condensates (arrowheads) over time in AML12 cells expressing FGFBP1-EGFP. Scale bar = 5 µm. **d** Representative micrographs of FGFBP1-EGFP condensates before and after photobleaching. Fluorescence recovery after photobleaching (FRAP) quantification of FGFBP1-EGFP condensates over a period of 3 min. Scale bar = 2.5 µm. **e** Schematic of the FGFBP1 domains and FGFBP1 mutants. **f** Fluorescence microscopy analysis of purified rFGFBP1-EGFP mutants (2 μmol/L, 10% PEG) mixed with heparin (10 μg/ml), and the size of the droplets was quantified. Scale bar = 5 µm. **g** HEK 293T cells were transfected with pCDH-CMV-FGFBP1 (WT, ΔHBS, ΔIDR1, or ΔIDR2)-Flag-EF1α-EGFP for 24 h and treated with or without heparin (5.5 μg/ml) for another 2 h. Western blotting analysis of flag levels in the cell culture medium and cell lysate. EGFP and α-tubulin levels in the cell lysate were used as a control. **h** Fluorescence microscopy analysis of purified rFGFBP1-EGFP (2 μmol/L, 10% PEG) mixed with rFGF6 (1 μmol/L) with or without heparin (10 μg/ml), and the size of the droplets was quantified. Scale bar = 5 µm. **i** HEK 293T cells were transfected with pCDH-CMV-FGFBP1-flag-EF1α-EGFP for 24 h and treated with rFGF6 (1 or 2 μmol/L) with or without heparin (5.5 μg/ml) for another 2 h. Western blotting analysis of flag levels in the cell culture medium and cell lysate was performed. EGFP and α-tubulin levels in the cell lysate were used as a control. **j** Concentration of FGFBP1 in the culture medium of differentiated primary myoblasts treated with or without heparin (5.5 μg/ml) and rFGF6 (1 or 2 μmol/L) (*n* = 3). **k** Proposed model for the regulation of FGFBP1 release from cell surface via LLPS. Image created with BioRender.com. ^*^P < 0.05, ^**^P < 0.01. FGFBP1 fibroblast growth factor binding protein 1, SP signal peptide, WT wild-type, HBS heparin-binding site, rFGF6 recombinant FGF6 protein, PEG polyethylene glycol, EGFP enhanced green fluorescent protein, pCDH-CMV plasmid cloning and delivery vector with cytomegalovirus promoter, EF1α elongation factor 1-alpha, HSPG heparan sulfate proteoglycan, HS heparan sulfate, FGF fibroblast growth factor, FGFRs fibroblast growth factor receptors

FGFBP1 bears two intrinsically disordered regions (IDRs) located at its N- (positions 24–63) and C- (positions 156–202) termini (Fig. 6a). We examined whether FGFBP1 could form condensates in vitro using proteins purified from *Escherichia coli*. Fluorescence microscopy analysis showed that enhanced green fluorescent protein (EGFP)-tagged FGFBP1 (FGFBP1-EGFP) readily self-associated as micrometer-sized spherical droplets in the absence of polyethylene glycol (PEG)-6000 (Fig. 6b). Consistent with the properties of liquids, FGFBP1 condensates dynamically, freely moves around, and fuses into larger particles that are immediately relaxed into a spherical structure (Fig. 6c). The fluorescence signals recovered 3 min after photobleaching (Fig. 6d), consistent with liquid-like condensates.

### Heparin promotes FGFBP1 release by attenuating phase separation

A previous study showed that heparin treatment increases FGFBP1 release from the cell membrane [42]; however, the underlying mechanism remains unknown. To test whether FGFBP1 phase separation is involved in this process, we generated several *Fgfbp1* deletion mutants, including the known heparin-binding site (ΔHBS) [43] and two IDRs (ΔIDR1 and ΔIDR2) (Fig. 6e), to determine whether FGFBP1 exhibits phase-separating behavior. Fluorescence microscopy showed that the HBS mutation did not affect condensate formation, whereas mutations in IDR1 or IDR2 significantly compromised FGFBP1 condensate formation in vitro. Notably, mixing heparin inhibited WT condensate formation, while it had no effect on the ∆HBS mutant (Fig. 6f). These data indicate that both IDRs are required for condensate formation and that heparin can inhibit condensate formation in the presence of the HBS domain.

To further clarify the involvement of phase separation on FGFBP1 release, we subcloned these mutations into a eukaryotic expression vector (pCDH-CMV-FGFBP1-Flag-EF1α-EGFP). Next, we transfected these vectors into human embryonic kidney 293T cells (HEK 293T) and treated them with or without heparin for 2 h at 24 h after transfection. The cells and medium were collected for analysis. Consistent with a previous study [42], the addition of heparin increased FGFBP1 levels in the culture medium and decreased FGFBP1 levels in the lysate. In contrast, FGFBP1 lacking the HBS could not be released, whereas almost all IDR-mutant FGFBP1 were released (Fig. 6g; Additional file 1: Fig. S12a). Importantly, we further determined the heparan sulfate levels in skeletal muscle after ALI and showed an increase and recovery pattern (Additional file 1: Fig. S12b, c). These results uncovered a unique mechanism for regulating the release of FGFBP1.

### FGF6 inhibits FGFBP1 release by competitively binding to heparin

FGF6 is a heparin-binding protein with multiple HBSs [44]. Recent data indicate that variants in the HBSs of FGF6 lead to an increase in free heparin levels [45]. Therefore, we hypothesized that FGF6 is involved in the formation of FGFBP1 condensates by competitively binding to heparin. To investigate this, we mixed rFGF6, heparin, or both with FGFBP1-EGFP in the absence of 10% PEG and examined the behavior of the mixture in vitro. We found that rFGF6 did not affect condensate formation compared to the control while restoring the inhibitory effect of heparin (Fig. 6h). To examine whether rFGF6 could inhibit FGFBP1 release in cells, we overexpressed FGFBP1 in 293T cells and collected the spent medium and cells as described above. The addition of rFGF6 decreased FGFBP1 levels in the medium in a dose-dependent manner (Fig. 6i**; **Additional file 1: Fig. S12d). Furthermore, we treated the differentiated primary myoblasts with heparin or heparin combined with rFGF6 to examine the release of endogenous FGFBP1. Consistently, heparin treatment increased FGFBP1 concentration in the medium, and rFGF6 reversed this effect (Fig. 6j). Collectively, our findings indicate that FGFBP1 forms condensate on the cell surface, and heparin negatively regulates condensate formation, affecting the homeostasis of FGFBP1 release and attachment (Fig. 6k).

Taken together, these results reveal a peripheral regulatory mechanism after ALI, in which elevated circulatory GCs target the skeletal muscle and induce the direct transrepression of *Fgf6*, resulting in attenuated protein synthesis, activation of *Fgfbp1* transcription, and increased FGFBP1 release. Hence, FGFBP1 acts as a messenger that promotes liver regeneration.

### FGF5 undergoes liquid–liquid phase separation (LLPS) and colocalization with FGFBP1 condensates

**Fig. 7:**
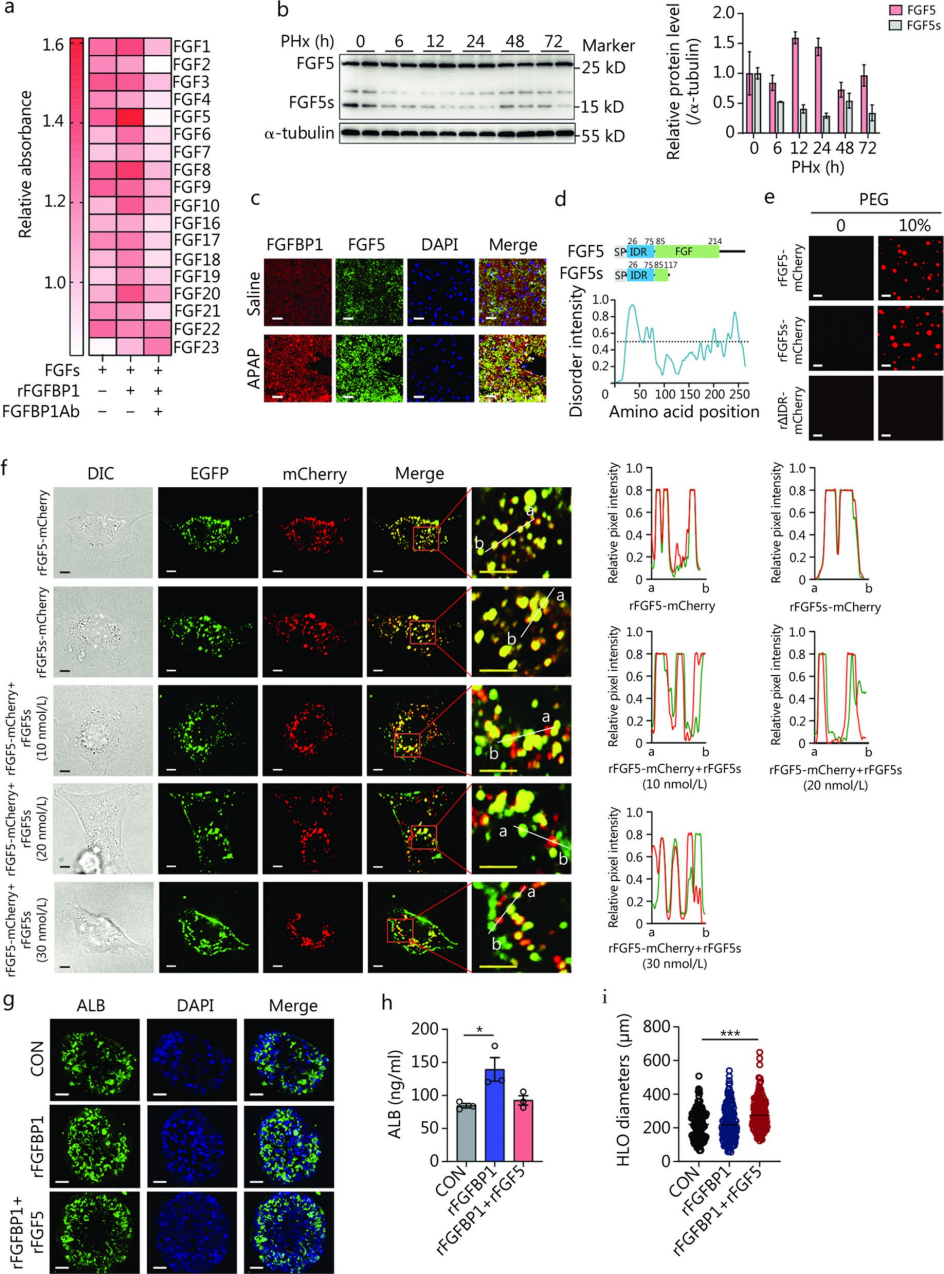
FGF5 and FGF5s compete for binding to FGFBP1 via LLPS and regulate liver regeneration.** a** BrdU cell proliferation assay results to detect cell proliferation ability in AML12 cells overexpressing the 18 FGFs and treated with rFGFBP1 (10 ng/ml) or rFGFBP1 and FGFBP1Ab (2 μg/ml) (*n* = 3). **b** Hepatic protein levels of FGF5 and FGF5s at the indicated times after partial (2/3) hepatectomy (PHx), with the corresponding quantification (*n* = 2). **c** Representative liver FGFBP1 and FGF5 immunofluorescence results 24 h after acetaminophen (APAP) or saline administration. **d** Domain structure and the intrinsically disordered tendency of mouse FGF5 and FGF5s. IUPred assigned scores of the disordered tendencies of the sequences between 0 and 1, with scores higher than 0.5 indicating disorder. The amino acid sequence of FGF5 contains one IDR. **e** Confocal microscopy images of the assembly status of 5 μmol/L purified rFGF5-mCherry, rFGF5s-mCherry, or rΔIDR-mCherry with 0 or 10% PEG. Scale bar = 5 µm. **f** Images of purified protein colocalization assay on the cell surface via confocal microscopy and representative curves describing the distribution of the relative fluorescence intensities of FGFBP1 (green) and FGF5 or FGF5s (red). Top two panels: rFGFBP1-EGFP (5 nmol/L) with rFGF5-mCherry (20 nmol/L) or rFGF5s-mCherry (10 nmol/L). Bottom three panels: rFGFBP1-EGFP (5 nmol/L) with rFGF5-mCherry (20 nmol/L) and different amounts of rFGF5s (10, 20, or 40 nmol/L). Scale bar = 5 µm. **g** Representative albumin (ALB, a hepatocellular function marker) immunofluorescence of human liver organoids (HLOs) in the indicated groups. **h** Measurement of ALB secretion in HLOs (*n* = 3). **i** Size calculation of HLOs in the indicated groups (CON, *n* = 146; rFGFBP1, *n* = 157; rFGFBP1 + rFGF5, *n* = 184). ^*^P < 0.05, ^***^P < 0.001. FGF5 fibroblast growth factor 5, FGF5s short form of FGF5, FGFBP1 fibroblast growth factor binding protein 1, LLPS liquid–liquid phase separation, IDR intrinsically disordered region, PEG polyethylene glycol, EGFP enhanced green fluorescent protein, DIC differential interference contrast, DAPI 4’,6-diamidino-2-phenylindole, SP signal peptide

To determine the potential FGFs that interact with FGFBP1 and promote hepatocyte proliferation, we overexpressed 18 FGFs in the murine hepatocyte cell line AML12, respectively, and treated the cells with recombinant FGFBP1 protein (rFGFBP1), FGFBP1Ab, or both. Cell proliferation assays identified FGF5 as a potential FGFBP1-binding protein (Fig. 7a). *Fgf5* encodes two protein isoforms, the long form (FGF5) and the short form (FGF5s), both of which have been proposed to serve as important regulators of hair length in mammals [46–48]. Notably, we observed that liver FGF5 levels were tightly associated with ALI, as FGF5 showed increased expression in the liver after PHx or AILI, whereas FGF5s expression was down-regulated (Fig. 7b; Additional file 1: Fig. S13a). In addition, immunofluorescence assays showed that FGFBP1 was distributed in a punctate pattern and colocalized with FGF5 in areas with APAP-induced damage (Fig. 7c). Subsequent bioinformatics analysis revealed an IDR at the N-terminus of FGF5 and FGF5s (Fig. 7d). Indeed, the purified FGF5-mCherry and FGF5s-mCherry proteins formed condensates in the absence of PEG in vitro, whereas the *Fgf5* mutant with IDR deletion (ΔIDR-mCherry) failed to form condensates (Fig. 7e). The condensate fusion and fluorescence recovery after photobleaching (FRAP) assays of exogenously expressed FGF5-mCherry in cells further indicated that FGF5 and FGF5s form LLPS (Additional file 1: Fig. S13b, c). Furthermore, both FGF5 and FGF5s showed good colocalization with FGFBP1 in cells and in vitro (Additional file 1: Fig. S13d, e). These results suggest that the clusters of the two proteins were likely condensed liquid-phase droplets.

### FGF5 and FGF5s compete for binding to FGFBP1 and regulate liver regeneration

Intriguingly, previous studies have shown that FGF5 and FGF5s competitively bind to cell surface receptors, while FGF5s act as a fake that does not efficiently activate the receptor, resulting in antagonized FGF5 activity [49, 50]. We then investigated whether this antagonism also limited the interaction between FGFBP1 and FGF5 in LLPS. We mixed recombinant FGF5s protein (rFGF5s) without fluorescent protein fusion with FGFBP1-EGFP and FGF5-mCherry. The presence of FGF5-mCherry in these clusters was diminished (Additional file 1: Fig. S13f), indicating that FGF5s can compete with FGF5 for binding to the FGFBP1 clusters. To directly examine the condensates on the cell surface, we added the purified proteins to the culture medium of AML12 cells. We observed that these proteins aggregated around the cell as condensates, whereas ΔIDR-mCherry did not (Additional file 1: Fig. S13g). Moreover, both FGF5-mCherry and FGF5s-mCherry colocalized with FGFBP1-EGFP and formed condensates on the cell surface, respectively (Fig. 7f, top two panels). Importantly, the addition of rFGF5s diminished FGF5-FGFBP1 condensation in a dose-dependent manner (Fig. 7f, bottom three panels). Functionally, we generated the human liver organoids (HLOs) by using human pluripotent stem cells (hPSCs) and treated them with rFGFBP1 or rFGFBP1 + rFGF5. Interestingly, rFGFBP1 treatment displayed enhanced function of HLOs, as evidenced by albumin (ALB) staining and supernatant ALB concentration, while having no significant effect on the size of HLOs (Fig. 7g–i). Of note, the size of HLOs was increased when treated with rFGFBP1 + rFGF5 (Fig. 7i). These results suggest a distinctive role of FGFBP1-FGF5 couple in promoting human liver cell proliferation.

**Fig. 8:**
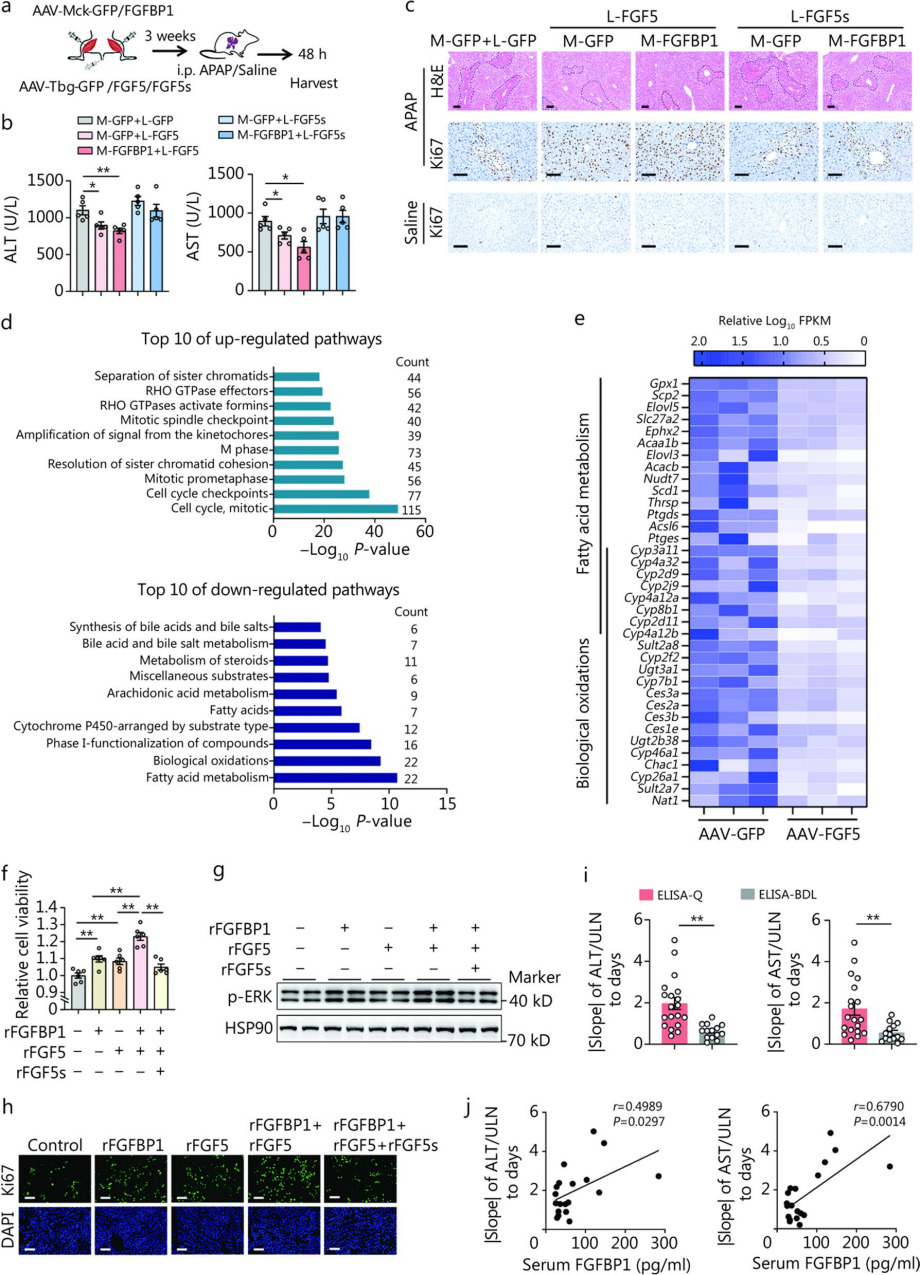
FGFBP1, FGF5, and FGF5s combination therapy on liver regeneration of acute liver injury (ALI) mice, and the association of serum FGFBP1 with the recovery of patients after ALI. **a** Schematic of the animal experiments. AAV-Mck-FGFBP1 (M-FGFBP1) or AAV-Mck-GFP (M-GFP) were administered to the Gas and Ta depots of 8-week-old C57BL/6 mice, and then AAV-Tbg-FGF5 (L-FGF5), AAV-Tbg-FGF5s (L-FGF5s), or AAV-Tbg-GFP (L-GFP) were injected into the tail vein. Acetaminophen (APAP) or saline dosing was performed 3 weeks later, and the livers and Gas were analyzed 48 h post-APAP or saline dosing. **b** Serum concentrations of alanine aminotransferase (ALT) and aspartate aminotransferase (AST) (*n* = 5). **c** Representative liver H&E (necrotic areas circled with black lines) and Ki67 (for indicating the proliferating cells) staining. Scale bar = 50 μm. **d** Reactome enrichment analysis of gene transcripts up-regulated and down-regulated in FGF5 overexpression livers, with top 10 terms shown. **e** Heatmap showing the genes of top 2 down-regulated pathways. Values are present as log_10_ FPKM relative to the average value of each gene in the control group. **f** Cell viability of AML12 cells treated with the indicated recombinant protein for 24 h as detected using a cell counting kit-8 (CCK-8) assay (*n* = 6). **g** Western blotting analysis of p-ERK expression in AML12 cells treated with the indicated recombinant proteins (*n* = 3). **h** Ki67 immunofluorescence in AML12 cells 24 h after treatment with the indicated recombinant proteins. **i** The decline rate of serum ALT and AST in ALI patients of the serum FGFBP1 between the groups of ELISA-Q and ELISA-BDL (*n* = 19 and 14, respectively). **j** The correlation between serum FGFBP1 and |slope| of ALT/ULN to days or |slope| of AST/ULN to days (*n* = 19). The data (**i**) were log-transformed and then analyzed using an unpaired Student’s *t*-test. ^*^P < 0.05, ^**^P < 0.01. FGF5 fibroblast growth factor 5, FGF5s short form of FGF5, FGFBP1 fibroblast growth factor binding protein 1, H&E hematoxylin and eosin, FPKM fragments per kilobase of transcript per million fragments mapped, ULN upper limit of normal, ELISA-Q enzyme-linked immunosorbent assay-quantifiable, ELISA-BDL enzyme-linked immunosorbent assay-below detection limit, AAV adenovirus-associated virus, Mck muscle creatine kinase, GFP green fluorescent protein, DAPI 4’,6-diamidino-2-phenylindole, Gas gastrocnemius, Ta tibialis anterior

To confirm this in vivo, we delivered AAV mediating the skeletal muscle-specific overexpression of GFP or FGFBP1 and the liver-specific overexpression of GFP, FGF5, or FGF5s in mice, which were then subjected to AILI 3 weeks after AAV injection (Fig. 8a). The ectopic expression of FGFBP1 and FGF5 attenuated AILI and promoted liver regeneration, whereas no significant effect was found in FGF5s-overexpressing mice (Fig. 8b, c). Consistently, we treated PHx mice with rFGFBP1 + rFGF5, rFGFBP1 + rFGF5 + rFGF5s, or PBS (Additional file 1: Fig. S14a). Evaluating the proliferative parameters revealed that rFGFBP1 + rFGF5 treatment largely promoted liver regeneration, whereas these benefits were blunted upon treatment with rFGF5s (Additional file 1: Fig. S14b–d). Further RNA-seq analysis identified a total of 1291 DEGs (800 up-regulated, 491 down-regulated) in FGF5 overexpression vs. GFP, using FDR < 0.05 and |log_2_ FC|> 1. Pathway enrichment analysis of DEGs revealed that FGF5 may improve AILI via regulating hepatic fatty acid metabolism and reducing lipid oxidative stress (Fig. 8d, e). Moreover, rFGF5 combined with rFGFBP1 significantly increased cell viability, phosphorylated ERK (p-ERK) levels, and the proportion of Ki67^+^ nuclei, whereas these effects were noticeably attenuated by rFGF5s (Fig. 8f–h**; **Additional file 1: Fig. S14e). Overall, these results demonstrate the potential contribution of FGFBP1-FGF5 condensates in enhancing liver regeneration after ALI and that FGF5s may serve as moderators to avoid abnormal cell proliferation.

### Serum FGFBP1 is beneficial for the recovery of patients after ALI

Of human relevance, we performed a clinical study to explore the impact of serum FGFBP1 concentration on the recovery of patients after ALI. A total of 33 patients who suffered from viral or drug-induced ALI were included (Additional file 1: Fig. S15a). Among them, 19 patients showed a detectable serum FGFBP1 (ELISA-Q, enzyme-linked immunosorbent assay-quantifiable), while 14 patients were below the detection limit of the ELISA kit (ELISA-BDL, enzyme-linked immunosorbent assay-below detection limit) (Additional file 1: Fig. S15a). We conducted a detailed analysis of the clinical characteristics, such as age, sex, and etiology of liver injury, across the patients (Additional file 1: Fig. S15b–g; Additional file 1: Table S1). The results revealed a negative correlation trend between initial serum liver enzyme levels and age, though it did not reach statistical significance (ALT, *P* = 0.0525; AST, *P* = 0.1596; Additional file 1: Fig. S15b). Additionally, no significant differences were found between the initial serum liver enzyme levels and sex or etiological factors among the enrolled patients (Additional file 1: Fig. S15c, d). Further subgroup analysis based on the detectability of serum FGFBP1 showed no significant differences in age, sex, or etiological factors distribution between the two groups (Additional file 1: Fig. S15e–g). Then, we calculated the decline rate |slope| of ALT and AST after hospitalization for each patient and analyzed the effect of serum FGFBP1 on the |slope|. As shown in Fig. 8i, the |slope| of the ELISA-Q group was significantly higher than the ELISA-BDL group, indicating patients with high serum FGFBP1 recover faster after ALI. Furthermore, we found that there is a close correlation between serum FGFBP1 with |slope| of ALT and AST, respectively, in patients of the ELISA-Q group (Fig. 8j). These results indicate the potential contribution of FGFBP1 in the repair of ALI in humans and that elevating FGFBP1 may serve as a promising target in treating ALI.

## Discussion

**Fig. 9:**
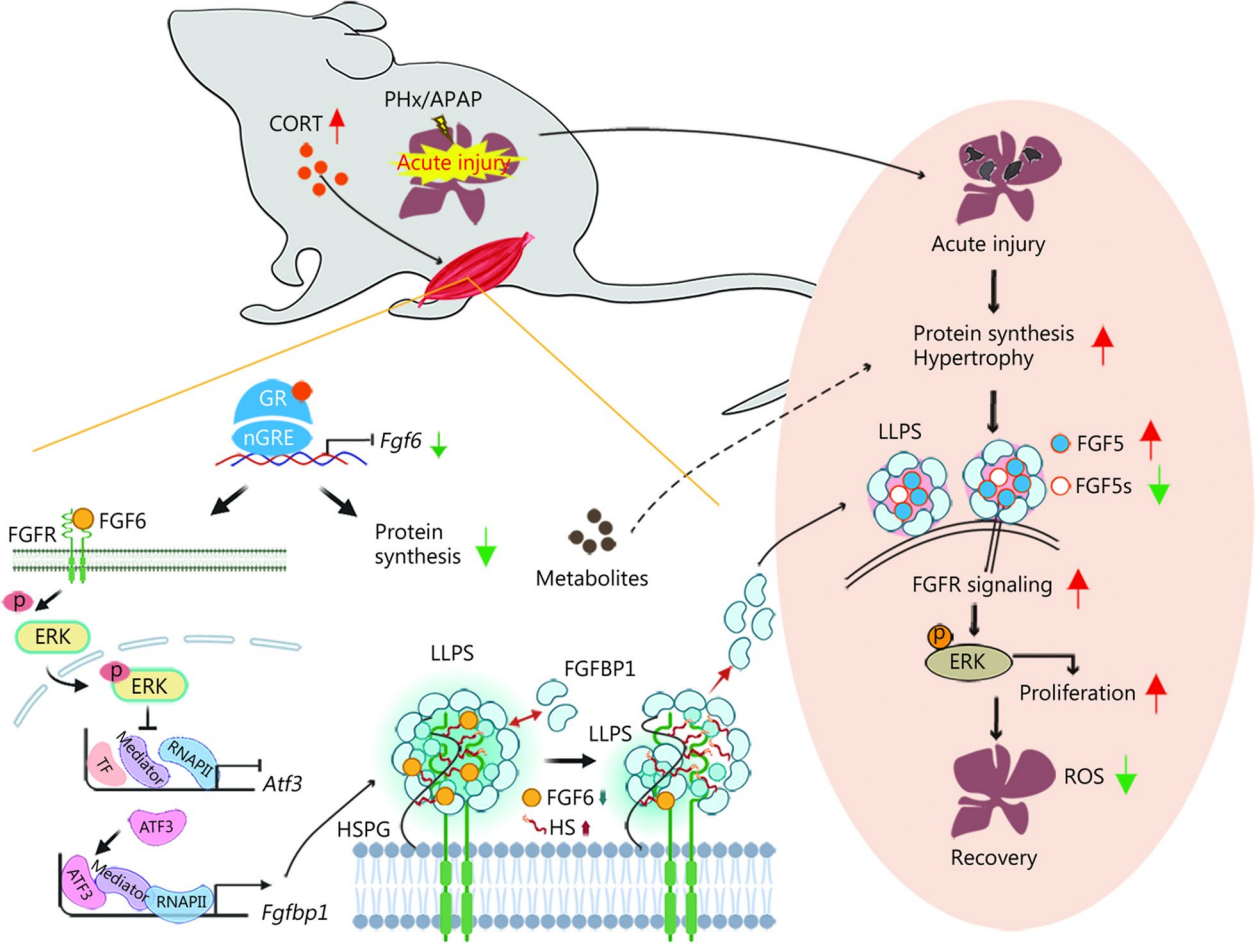
Illustration summarizing the muscle-liver crosstalk in protecting against ALI and promoting liver regeneration. PHx or APAP-induced ALI elevates CORT, which activates muscle GR to suppress *Fgf6* transcription. This suppression triggers two downstream effects: 1) inhibits protein synthesis and enhances catabolism, potentially providing substrates for pre-regenerative hepatocyte hypertrophy; and 2) FGF6 regulates *Fgfbp1* transcription via the ERK-ATF3 axis. FGFBP1 forms membrane-associated condensates through LLPS. Free HS antagonizes LLPS to promote FGFBP1 release. FGF6 sequesters HS to stabilize LLPS, while FGF6 depletion liberates HS, disrupting LLPS and enhancing FGFBP1 secretion. Circulating FGFBP1 interacts with hepatic FGF5/FGF5s via LLPS to coordinate liver regeneration. CORT corticosterone, PHx partial (2/3) hepatectomy, APAP acetaminophen, GR glucocorticoid receptor, nGRE negative GC response elements, FGF6 fibroblast growth factor 6, FGFR fibroblast growth factor receptor, ERK extracellular signal-regulated kinases, ATF3 activating transcription factor 3, TF transcriptional factor, RNAP II RNA polymerase II, FGFBP1 fibroblast growth factor binding protein 1, LLPS liquid–liquid phase separation, HSPG heparan sulfate proteoglycan, HS heparan sulfate, FGF5 fibroblast growth factor 5, FGF5s short form of FGF5, ROS reactive oxygen species

In this study, we demonstrate that GCs exert key metabolic actions in the skeletal muscle after ALI, which trigger muscle-liver crosstalk to protect against ALI and promote liver regeneration through the FGF6-FGFBP1 axis (Fig. 9). GCs are commonly used to treat ALI, but their clinical outcomes remain controversial [25]. As inflammatory factors are critical for initiating liver regeneration [51], and Dex treatment inhibits the expression of inflammatory cytokines interleukin (IL)-6 and tumor necrosis factor-α during liver regeneration, thereby impeding hepatocyte proliferation [23], suggesting that the timing of exogenous GCs administration may significantly affect therapeutic outcomes. We show that elevated levels of endogenous GCs induced by ALI target the peripheral skeletal muscles and are essential for liver regeneration. However, further administration of low-dose Dex to the skeletal muscle did not provide additional benefit to liver regeneration. These results indicate that the elevated levels of endogenous GCs during ALI in healthy individuals are satiable for initiating liver regeneration. In addition, given that IL-6 is recognized as a myokine [52], the use of GCs should also consider the systemic levels of key inflammatory cytokines involved in liver regeneration within the circulatory system. Importantly, *Fgf6* deficiency or FGF6Ab treatment in the skeletal muscle further promoted liver regeneration, suggesting that FGF6 as the downstream target of GCs is more effective for the treatment of ALI. However, under stress conditions, significant differences exist between male and female animals in stress responses and endocrine-metabolic regulation [53]. These differences notably include variations in GR signaling regulation and differential expression of FGFs [54–56]. The present study was limited to male mice and thus data regarding the sex-dependent metabolic difference between males and females during ALI remain elusive.

LLPS, a dynamic and reversible process that drives the formation of membraneless condensates, has been recognized to play critical roles in multiple biological processes [57, 58]. Our data demonstrated that FGFBP1 underwent LLPS, which is in line with its reported characteristics: exhibiting a noncovalent and reversible binding form and enhanced activity at low ligand concentrations [41]. We established both prokaryotic and eukaryotic overexpression systems for FGFBP1 and its mutants to compare the formation of LLPS and its release. Our data suggest that FGFBP1 binds to the cell membrane in a phase separation-dependent manner and that heparin disturbs the phase separation that promotes FGFBP1 release. Notably, mutation of HBS in FGFBP1 abolished the inhibitory effect of heparin and disrupted the secretion of the protein into the culture supernatant. These results indicated that FGFBP1 may mainly bind to cell surface receptors other than heparan sulfate proteoglycan. Importantly, FGFBP1 exists in the supernatant under normal non-heparin treatment, which suggests that endogenous heparin and FGFBP1 exist in a dynamic balance and that the release of FGFBP1 maintains sensitivity to heparin.

FGFBP1 has been reported to bind FGF1 and FGF2 and enhance biological activities in carcinoma cells [41, 59]. Follow-up studies demonstrated that FGFBP1 enhances wound repair and muscle reinnervation by interacting with FGF7, FGF10, and FGF22 [42, 60, 61]. Besides, several FGFs have been demonstrated to be involved in regulating liver regeneration, such as FGF2 [62], FGF7 [63] and FGF15 [64]. Our screening system using normal liver cell lines identified FGF5 as a new partner of FGFBP1 in promoting hepatocyte proliferation. Of note, we found that the mRNA level of *Fgf5* in the liver was undetectable via qRT-PCR, while it has a considerable protein level as shown by Western blotting and immunohistochemistry. These results are consistent with another characteristic of LLPS, which is locally concentrating molecules in condensates to activate reactions and signaling processes [57]. Interestingly, the FGF5s, which have the IDR retained but lost the FGF domain, can form condensates efficiently and compete with FGF5 for colocalization with FGFBP1 condensates. This explains FGF5s as an antagonist of FGF5 activity in neurotrophic activity in the brain and hair growth, as previously reported [48, 49], as well as liver regeneration. Moreover, the opposite protein expression patterns of FGF5 and FGF5s in the liver after ALI suggest an alternative mRNA splicing regulation, which will require further study.

HLOs induced by hPSCs have recently been used as a model for studying ALI and liver regeneration [30, 65]. An interesting observation of this study is that low-dose (5–25 ng/ml) FGFBP1 treatment induced an increased expression of ALB in HLOs, while the combination of FGFBP1 and FGF5 promoted HLO proliferation. These results confirmed the effective proliferative effect of FGFBP1 combined with FGF5 on liver regeneration. More importantly, our preliminary clinical study revealed the association of serum FGFBP1 with the recovery of patients after ALI, and our data indicate that high serum FGFBP1 is beneficial to the recovery of ALI. Of note, nearly half of the serum samples (42.4%) exhibited FGFBP1 levels below the detection limit (23.4 pg/ml) for ELISA, suggesting that FGFBP1 may hold significant therapeutic potential in ALI. However, we do not know the reason for the low level of serum FGFBP1 in these patients, such as the transcriptional regulation or release blockade.

## Conclusions

Our study identified a direct GC transrepression target in the skeletal muscle, FGF6, that potently regulates skeletal muscle metabolic activity, and controls FGFBP1 transcription and release during ALI. Subsequently, circulatory FGFBP1 integrates with hepatic FGF5 via an LLPS manner and promotes liver regeneration. Importantly, our preliminary clinical study revealed the association of serum FGFBP1 with the recovery of patients with ALI. Overall, our results demonstrate a molecular mechanism by which muscle-liver crosstalk can initiate and sustain liver regeneration, providing a potential therapeutic target and treatment strategy for ALI.

## Supplementary Information

**Additional file 1. Methods. Table S1** Patient demographics and clinical characteristics. **Table S2** The primers used in this study. **Fig. S1** Tissue weight and Western blotting analysis of the protein synthesis rate of mice 12 h after PHx or sham surgery. **Fig. S2** Dexamethasone (Dex) treatment inhibits fibroblast growth factor 6 (FGF6) expression, and acetaminophen (APAP)-induced acute liver injury activates GCs signaling transduction. **Fig. S3** Skeletal muscle specific-delivery of si*Nr3c1* has no effect on the level of GR in the liver. **Fig. S4** Skeletal muscle administration of nandrolone or mifepristone impaired liver regeneration. **Fig. S5** Intramuscularly administered low-dose dexamethasone (Dex) has no effect on liver regeneration after acute liver injury (ALI). **Fig. S6** Skeletal muscle overexpression of FGF6 inhibits liver regeneration after PHx in mice. **Fig. S7** *Fgf6* deficiency promotes liver regeneration after PHx. **Fig. S8** Determination of the FGF6 targets secretory factors in Gas muscles. **Fig. S9** FGF6 suppresses the transcription of *Fgfbp1* via ERK-ATF3 axis in muscle. **Fig. S10** The effects of skeletal muscle injection with AAV-Mck-FGFBP1 or AAV-Mck-GFP. **Fig. S11** Blocking FGFBP1 specifically in the skeletal muscle of *Fgf6*-KO mice abolished the protective effects in these mice upon PHx. **Fig. S12** Dynamic changes of heparan sulfate in muscle after acute liver injury (ALI). **Fig. S13** FGF5 undergoes LLPS and colocalization with FGFBP1 condensates. **Fig. S14** FGFBP1, FGF5, and FGF5s combination therapy on liver regeneration of acute liver injury (ALI) mice. **Fig. S15** Clinical characteristics of patients with acute liver injury (ALI).

**Additional file 2.** RNA-seq and ATAC-seq analysis data of the Gas from the Sham group and PHx group at 12 h post-treatment.

**Additional file 3.** RNA-seq analysis data of the Gas from the GFP control group and the FGF6 overexpression group.

## Data Availability

All data supporting the conclusions in this manuscript can be found in the main text or the supplementary materials. RNA-Seq data and ATAC-seq data were deposited to the NCBI Sequencing Read Archive (SRA) under accession numbers PRJNA1177399, PRJNA1131661, and PRJNA1131737.

## Abbreviations

ALI: Acute liver injury
APAP: Acetaminophen
AAV: Adenovirus-associated virus
ATF3: Activating transcription factor 3
ALB: Albumin
ALT: Alanine aminotransferase
AILI: APAP-induced liver injury
AST: Aspartate aminotransferase
ATAC-seq: Assay for transposase-accessible chromatin by sequencing
BAX: BCL2 associated X
BAT: Brown adipose tissue
ChIP: Chromatin immunoprecipitation
ChIP-qPCR: Chromatin immunoprecipitation quantitative PCR
Cl-CASP3: Cleaved caspase-3
CORT: Corticosterone
DEGs: Differentially expressed genes
Dex: Dexamethasone
DKK3: Dickkopf WNT signaling pathway inhibitor 3
ELISA: Enzyme-linked immunosorbent assay
ELISA-BDL: Enzyme-linked immunosorbent assay-below detection limit
ELISA-Q: Enzyme-linked immunosorbent assay-quantifiable
eWAT: Epididymal white adipose tissue
EGFP: Enhanced green fluorescent protein
ERK: Extracellular signal regulated kinase
FDR: False discovery rate
FBS: Fetal bovine serum
FGF5: Fibroblast growth factor 5
FGF6: Fibroblast growth factor 6
FGFBP1: Fibroblast growth factor binding protein 1
FGF6Ab: FGF6-neutralizing antibody
FRAP: Fluorescence recovery after photobleaching
FPKM: Fragments per kilobase of transcript per million fragments mapped
GR: GC receptor
Gas: Gastrocnemius
GO: Gene Ontology
GCs: Glucocorticoids
GFP: Green fluorescent protein
HSP90: Heat shock protein 90
H&E: Hematoxylin and eosin
HBS: Heparin-binding site
HEK 293T: Human embryonic kidney 293T cells
HLOs: Human liver organoids
hPSCs: Human pluripotent stem cells
iWAT: Inguinal white adipose tissue
ITS: Insulin–transferrin–sodium
IDRs: Intrinsically disordered regions
IL: Interleukin
IPTG: Isopropyl-beta-thiogalactopyranoside
KO: Knockout
LLPS: Liquid–liquid phase separation
Mck: Muscle creatine kinase
nGREs: Negative GC response elements
Nr3c1: Nuclear receptor subfamily 3 group c member 1
PHx: Partial (2/3) hepatectomy
PCDH: Plasmid cloning and delivery vector with hygromycin
P/S: Penicillin/streptomycin
PBS: Phosphate-buffered saline
p-ERK: Phospho-extracellular signal regulated kinases
PCA: Principal component analysis
PCNA: Proliferating cell nuclear antigen
PEG: Polyethylene glycol
Qu: Quadriceps
qRT-PCR: Quantitative real-time PCR
rFGF6: Recombinant FGF6 protein
rFGFBP1: Recombinant FGFBP1 protein
rFGF5: Recombinant mouse FGF5 protein
rFGF5s: Recombinant mouse FGF5s protein
RIN: RNA integrity number
RNA-seq: RNA-sequencing
siNC: Scramble controls
FGF5s: Short form of FGF5
Sol: Soleus
SD: Standard deviation
SEM: Standard error of the mean
SUnSET: Surface sensing of translation
TUNEL: Terminal deoxynucleotidyl transferase-mediated deoxyuridine triphosphate nick-end labeling
Ta: Tibialis anterior
Tbg: Thyroxine-binding globulin
VTN: Vitronectin
WT: Wild-type
