# Supplementary material for: Glucocorticoids trigger muscle-liver crosstalk to attenuate acute liver injury and promote liver regeneration via the FGF6-FGFBP1 axis
Source: Mil Med Res. 2025 Jul 21;12:36. doi: 10.1186/s40779-025-00618-y (PMC12278513; doi:10.1186/s40779-025-00618-y)
Supplement: Supplementary file 1 — Additional file 1. Methods. Table S1 Patient demographics and clinical characteristics. Table S2 The primers used in this study. Fig. S1 Tissue weight and Western blotting analysis of the protein synthesis rate of mice 12 h after PHx or sham surgery. Fig. S2 Dexamethasone (Dex) treatment inhibits fibroblast growth factor 6 (FGF6) expression, and acetaminophen (APAP)-induced acute liver injury activates GCs signaling transduction. Fig. S3 Skeletal muscle specific-delivery of siNr3c1 has no effect on the level of GR in the liver. Fig. S4 Skeletal muscle administration of nandrolone or mifepristone impaired liver regeneration. Fig. S5 Intramuscularly administered low-dose dexamethasone (Dex) has no effect on liver regeneration after acute liver injury (ALI). Fig. S6 Skeletal muscle overexpression of FGF6 inhibits liver regeneration after PHx in mice. Fig. S7 Fgf6 deficiency promotes liver regeneration after PHx. Fig. S8 Determination of the FGF6 targets secretory factors in Gas muscles. Fig. S9 FGF6 suppresses the transcription of Fgfbp1 via ERK-ATF3 axis in muscle. Fig. S10 The effects of skeletal muscle injection with AAV-Mck-FGFBP1 or AAV-Mck-GFP. Fig. S11 Blocking FGFBP1 specifically in the skeletal muscle of Fgf6-KO mice abolished the protective effects in these mice upon PHx. Fig. S12 Dynamic changes of heparan sulfate in muscle after acute liver injury (ALI). Fig. S13 FGF5 undergoes LLPS and colocalization with FGFBP1 condensates. Fig. S14 FGFBP1, FGF5, and FGF5s combination therapy on liver regeneration of acute liver injury (ALI) mice. Fig. S15 Clinical characteristics of patients with acute liver injury (ALI). [file 40779_2025_618_MOESM1_ESM.pdf]

Methods

Adenovirus-associated virus (AAV) and siRNA delivery

The double muscle creatine kinase (*Mck*) promoter was used to induce skeletal muscle-specific AAV2/9-mediated gene transfer for fibroblast growth factor 6 (FGF6), fibroblast growth factor binding protein 1 (FGFBP1), and green fluorescent protein (GFP) overexpression (Hanbio Biotechnology Co., Ltd., Shanghai, China). Briefly, mice were anesthetized using isoflurane (5% for induction and 2% for maintenance). The AAVs were then directly injected into the gastrocnemius (Gas) and tibialis anterior (Ta) on both sides (2×10^{10} plaque-forming units). For liver-specific overexpression, hepatocyte-specific thyroxine-binding globulin (*Tbg*) promoter-driven AAV2/8 expressing FGF5, short form of FGF5 (FGF5s), or GFP (AAV-Tbg-FGF5, AAV-Tbg-FGF5s, and AAV-Tbg-GFP, respectively) were generated by Hanbio (Shanghai, China). Next, 8-week-old C57BL/6J mice were injected with AAV-Tbg-FGF5, AAV-Tbg-FGF5s, or AAV-Tbg-GFP (1×10^{11} plaque-forming units) via tail vein.

For the glucocorticoid receptor (GR) knockdown experiments, we delivered a mix of 4 pre-validated siRNAs, as previously reported [1] against mouse *Nr3c1* (encoding GR) in the Gas and Ta, using a commercial kit (Entranster-in vivo; Engreen Biosystem Co., Ltd., Beijing, China) [2].

Chemical compounds, recombinant protein, and neutralizing antibody administration

Water-soluble dexamethasone (Dex) phosphate disodium (T0947L, TargetMol, Shanghai, China) was used to treat the acute liver injury (ALI) mice. Specifically, mice were subjected to partial (2/3) hepatectomy (PHx) or acetaminophen (APAP) dosing and injected with Dex (50 μg/kg body weight) 2 h after ALI. The mice were sacrificed at the indicated times. Mifepristone (T1102, TargetMol, Shanghai, China) and nandrolone phenylpropionate (T8709, TargetMol, Shanghai, China) need to be prepared as stock solutions using DMSO and then diluted into an injectable solution using polyethylene glycol (PEG)-300, Tween 80, and ultrapure water. The solution is administered via continuous intramuscular injection (1 mg/kg body weight) for 3 d. PHx is performed 2 h after the second injection, and samples are collected 48 h after the PHx.

For recombinant mouse FGF6 protein (rFGF6; 5750-F6, R&D Systems, MN, USA) administration to the skeletal muscle, the mice were anesthetized with isoflurane (5% for induction and 2% for maintenance), and rFGF6 or phosphate-buffered saline (PBS) was directly injected into the

Gas and Ta on both sides (2 μg per mouse). For the intraperitoneal (i.p.) injection of recombinant rat FGFBP1 protein (rFGFBP1; 1413-FB, R&D Systems, MN, USA), recombinant mouse FGF5 protein [rFGF5; *Escherichia coli* (*E. coli*)-derived and purified in this study], or recombinant mouse FGF5s protein (rFGF5s; *E. coli*-derived and purified in this study), mice were subjected to PHx and i.p. injection of PBS, rFGFBP1 (1 μg/kg) + rFGF5 (20 μg/kg), or rFGFBP1 (1 μg/kg) + rFGF5 (20 μg/kg) + rFGF5s (40 μg/kg) at 8 and 24 h after PHx. The mice were then sacrificed 48 h after PHx.

For neutralizing antibody delivery, the mice were anesthetized using isoflurane (5% for induction and 2% for maintenance) before administration. Then, FGF6-neutralizing antibody (FGF6Ab; AB-238-NA, R&D Systems, MN, USA) or IgG (AB-108-C, R&D Systems, MN, USA) were directly injected into the Gas and Ta on both sides of the mouse (2 mg/kg body weight) at the indicated times after PHx and APAP dosing. In addition, FGFBP1-neutralizing antibody (FGFBP1Ab; AF1413, R&D Systems, MN, USA) or IgG (AB-108-C, R&D Systems, MN, USA) were directly injected into the Gas and Ta on both sides of the mice (0.5 mg/kg body weight) at the indicated time for PHx.

Western blotting analysis

Tissues or cells were lysed with RIPA buffer (Beyotime Technology, Shanghai, China) containing protease and phosphatase inhibitors (Roche, Mannheim, Germany) on ice and then centrifuged at 12,000 rpm for 15 min. The supernatants were collected and the total protein concentration was quantified using a BCA Protein Assay Kit (Beyotime Technology Shanghai, China). Next, 5 μg/μl protein extract was added to the loading buffer (Beyotime Technology, Shanghai, China) and denatured by boiling at 95 °C for 10 min. Equal amounts of total protein were then separated via SDS-PAGE, transferred onto nitrocellulose membranes (Merck Millipore, MA, USA), and blocked with 5% skim milk (Cell Signaling Technology, Danvers, MA, USA) for 1 h at room temperature. After incubation with the desired antibodies overnight at 4 °C, the membranes were washed in TBST (Tris-buffered saline containing 0.1% Tween 20) 3 times, followed by incubation with the corresponding horseradish peroxidase-conjugated secondary antibodies (HRP-linked anti-rabbit IgG, 7074, Cell Signaling Technology, MA, USA, or HRP-linked anti-mouse IgG, 7076, Cell Signaling Technology, MA, USA) for 1 h at room temperature. Protein bands were visualized using an ECL chemiluminescence kit (Merck Millipore, MA, USA) on a ChemiDoc Touch Imaging System (Bio-Rad, CA, USA). Image Lab software (version 6.0) was used to acquire images of the blots. ImageJ software version 1.52

(National Institutes of Health) was used for Western blotting image densitometry measurements. The following primary antibodies were used: GR (12041S, Cell Signaling Technology, MA, USA), FGF6 (D262668, Sangon Biotech, Shanghai, China), FGFBP1 (25006-1-AP, ProteinTech, Wuhan, China), dickkopf WNT signaling pathway inhibitor 3 (DKK3; A22126, Abclonal, Wuhan, China), BCL2 associated X (BAX; 5023S, Cell Signaling Technology, MA, USA), cleaved caspase-3 (Cl-CASP3; 9664S, Cell Signaling Technology, MA, USA), cyclin D1 (GB111935, Servicebio, Wuhan, China), cyclin E (GB111936, Servicebio, Wuhan, China), proliferating cell nuclear antigen (PCNA; GB11010, Servicebio, Wuhan, China), FGF5 (ab88118, Abcam, Cambridge, UK), phospho-extracellular signal regulated kinases (p-ERK; 4370S, Cell Signaling Technology, MA, USA), heat shock protein 90 (HSP90; 4874S, Cell Signaling Technology, MA, USA), flag (14793S, Cell Signaling Technology, MA, USA), enhanced green fluorescent protein (EGFP; GB15603, Servicebio, Wuhan, China), and heparan sulfate (ZMS1091, Sigma, MO, USA).

Quantitative real-time PCR (qRT-PCR)

Total RNA was extracted from hepatic tissues or cells using TRIzol reagent (Invitrogen/Life Technologies, Carlsbad, CA, USA) and reverse-transcribed into cDNA using a PrimeScript RT reagent kit with gDNA Eraser (TaKaRa, Dalian, China). qRT-PCR analysis was performed with a SYBR Green Master Mix (Applied Biosystems, CA, USA) in a QuantStudio Real-Time PCR System (Applied Biosystems, CA, USA) according to the manufacturer's instructions. Relative mRNA expression was quantified according to the Pfaffl method after normalizing against the expression of housekeeping gene *18s* (gene expression profile), *36b4* (liver), or *Gapdh* (muscle, intestine, C2C12 cell). The primers used are listed in **Additional file 1: Table S2**.

Histologic analysis

Liver tissues were fixed overnight in 4% formalin, embedded in paraffin, and cut into 5-μm-thick sections. Paraffin-embedded tissue sections were then stained with hematoxylin and eosin (H&E) or analyzed via immunohistochemistry. Frozen sections were prepared for immunofluorescence analysis. For immunohistochemistry, the sections were deparaffinized, rehydrated, and incubated with primary antibodies against Ki67 (GB121141, Servicebio, Wuhan, China) or heparan sulfate (ZMS1091, Sigma, MO, USA), followed by incubation with the corresponding horseradish peroxidase-conjugated

secondary antibodies. The sections were then treated with a DAB Staining Kit (G1212, Servicebio, Wuhan, China) according to the manufacturer's instructions. The following antibodies were used for immunofluorescence analysis: FGFBP1 (25006-1-AP, ProteinTech, Wuhan, China), FGF5 (ab88118, Abcam, Cambridge, UK), albumin (ALB; MAB1455, R&D systems, MN, USA), and 4',6-diamidino-2-phenylindole (DAPI; C1002, Beyotime Biotechnology, Shanghai, China). Antigens were visualized using a tyramide signal amplification kit (G1226, Servicebio, Wuhan, China) according to the manufacturer's protocol. Images were acquired using a digital microscope (Leica, Wetzlar, Germany) and analyzed using ImageJ version 1.52 software (National Institutes of Health, USA).

Assessment of apoptosis using the terminal deoxynucleotidyl transferase-mediated deoxyuridine triphosphate nick-end labeling (TUNEL) assay

Paraffin sections were stained using a TUNEL assay kit (G1504, Servicebio, Wuhan, China). In brief, after deparaffinization and rehydration, the sections were rinsed with Dulbecco's phosphate buffered saline and treated with 0.8% proteinase K at room temperature for 15 min, and then incubated with a TUNEL reaction mixture for 1 h in a 37 °C humidified chamber in the dark. After washing, the sections were mounted with Vectashield mounting medium with DAPI and visualized under a fluorescence microscope (Leica, Wetzlar, Germany). Finally, the images were analyzed using ImageJ version 1.52 software (National Institutes of Health, USA).

RNA-sequencing (RNA-seq) and bioinformatics analysis

For RNA-seq, total RNA was extracted from Gas or liver samples using TRIzol (Invitrogen, CA, USA) according to the manufacturer's instructions. The concentration of RNA was assessed using a NanoDrop (ThermoFisher, MA, USA), and the quality was evaluated on an Agilent 2100 bioanalyzer (ThermoFisher, MA, USA). All samples with an RNA integrity number (RIN) ≥ 7.0 and a 28S/18S ratio ≥ 1.5 were used to prepare sequencing libraries to ensure high-quality sequencing data. RNA libraries were prepared using the VAHTS® Universal V6 RNA-seq Library Prep Kit for IlluminaVazyme (Cat. No. 401-02) and then sequenced using the Novaseq 6000 platform in a paired-end configuration. Quality control and preprocessing of the raw sequencing reads were conducted using RSeQC (version 2.6.4). Clean reads were obtained by removing reads containing a sequencing adapter, reads with a high ratio (more than 20%) of low-quality bases (base quality less than or equal

to 5), and reads with a high ratio (more than 5%) of unknown bases ("N" base) from the raw data. The clean reads for each sample were aligned to the Mus musculus reference genome using HISAT2 (v2.0.4). Gene expression levels were quantified in terms of the fragments per kilobase of transcript per million fragments mapped (FPKM) by RSEM (v1.2.12). No samples were excluded based on principal component analysis (PCA) and hierarchical clustering. Differentially expressed genes (DEGs) were identified using the RankProd49 package in R, based on false discovery rate (FDR) < 0.05 and $|\log_2$ fold change (FC)$| > 1$. Gene Ontology (GO) analysis (geneontology.org) was used to perform enrichment analysis on DEGs.

Measurement of corticosterone, FGFBP1, and ALB levels

Mouse plasma corticosterone was detected using a corticosterone enzyme-linked immunosorbent assay (ELISA) kit (E-OSEL-M0001, Elabscience, Wuhan, China). Serum FGFBP1 was measured using FGFBP1 ELISA kits (Human: DY1593, R&D Systems, MN, USA; Mouse: RK08068, Abclonal, Wuhan, China). Supernatant ALB of cultured human liver organoids (HLOs) was measured using ALB ELISA kits (E-TSEL-H0029, Elabscience, Wuhan, China).

Chromatin immunoprecipitation (ChIP) assays

ChIP assays were carried out using a SimpleChIP® Plus Enzymatic Chromatin IP Kit (9003, Cell Signaling Technology, MA, USA) following the manufacturer's protocol. Briefly, mouse Gas muscles (100 mg) were dissected, minced into small pieces, and cross-linked with 1% formaldehyde at room temperature for 10 min, followed by quenching with a 0.125 mol/L glycine solution. Cell suspensions were prepared via Dounce homogenization in ice-cold PBS. Chromatin fragmentation was performed via sonication in SDS lysis buffer using a Bioruptor (Scientz, Ningbo, China) to an approximate DNA size of 500 bp. The samples were then immunoprecipitated overnight at 4 °C with rabbit anti-GR antibody (12041, Cell Signaling Technology, MA, USA). Normal rabbit IgG (2729, Cell Signaling Technology, MA, USA) served as the negative control. Protein G Magnetic Beads were then added, and the samples were incubated for another 2 h at 4 °C. After standard washes, elution buffer was added to all the immunoprecipitated and input samples. DNA from each sample was purified using real-time PCR. The primer sequences used in this experiment are listed in **Additional file 1: Table S2**.

Luciferase reporter assays

HEK293T cells were cultured in high-glucose DMEM with 10% fetal bovine serum (FBS) and 1% penicillin/streptomycin (P/S) and transfected with plasmids using Lipofectamine 2000 reagent (Invitrogen, CA, USA), following the manufacturer's instructions. For the luciferase reporter assays, 200 ng of the reporter plasmid, 300 ng of the expression plasmid, and 10 ng of pRL-SV40 (Renilla luciferase control reporter vector) were co-transfected into HEK293T cells in 48-well plates. Cells were collected 36 h after transfection, and relative luciferase activity was measured using a Dual-Luciferase Reporter Assay System (Promega, WI, USA). Each experiment was performed in triplicate.

Assay for transposase-accessible chromatin by sequencing (ATAC-seq) analysis

Nuclei were extracted from frozen Gas muscle tissue, as previously described [3]. Briefly, 50 mg of frozen Gas tissue from three different samples was added to a pre-chilled 1 ml Dounce with 1 ml cold homogenization buffer, then subjected to homogenization with a loose pestle for 10 strokes and with a tight pestle for 25 strokes. After centrifugation to remove large cellular debris from the homogenized liver fluid, the supernatant containing the nuclei was further purified using gradient density centrifugation. Approximately 50,000 purified nuclei were used to generate an ATAC-seq library using a TruePrep DNA Library Prep Kit V2 for Illumina (TD501, Vazyme, Nanjing, China). The library was then purified using VAHTS DNA Clean Beads (N411, Vazyme, Nanjing, China) and sequenced using a 2 × 150 bp Illumina HiSeq platform.

For data analysis, paired-end reads from the ATAC-seq library were trimmed using Trim Galore (v0.6.7) and aligned to the reference mouse genome UCSC mm10 using Bowtie 2 (v2.2.5) with the default parameters. After removing duplicates, multiple mapping reads and reads mapped to the mitochondrial genome, high-quality reads were picked using the SAMtools (v1.7) view function with the following settings: "-f 1 -F 4 -F 8 -F 256 -F 2048 -q 30". To identify reads from the nucleosome-free region, fragments with insert sizes < 100 bp were extracted using SAMtools and awk. ATAC-seq peaks were called using the HOMER (v4.11) findPeaks function with the following parameters: "-style dnase -minDist 200 -size 100". Peak regions that overlapped the mm10 ENCODE exclusion list were discarded. For visualization, deepTools (v3.5.1) was used to normalize the ATAC-seq read counts to Counts Per Million values with the following parameters: "--normalizeUsing CPM -bs 50". The tag counts in each merged region were extracted and compared between groups. Differentially accessible regions between sham and PHx were identified using the R package DiffBind (v3.8.4), with the

following statistical cutoff: FDR (Benjamini and Hochberg correction) < 0.05 and $|\log_2 FC| > 1$. The average profiles of regions of interest in each group were determined using ngs.plot (v2.61) with the following parameters: "-L 3000 -FL 100 -MW 4 -SE 0".

Puromycin incorporation assay

Protein synthesis was measured using surface sensing of translation (SUnSET) [4,5]. Briefly, mice were intraperitoneally injected with 0.04 μmol/g body weight puromycin (MB2005-1, MeilunBio, Daliang, China), dissolved in 100 μl of PBS. Thirty minutes after injection, muscle samples were collected and frozen in liquid nitrogen. Puromycin levels were analyzed via Western blotting. The change in protein synthesis rate is calculated as the percentage difference between the treatment group and the control group, derived by subtracting the anti-puromycin protein band intensity of the control group from that of the treatment group, then dividing the result by the control group value and multiplying by 100%.

Biochemical analysis

Serum samples were assayed for alanine aminotransferase (ALT) and aspartate aminotransferase (AST) levels using commercially available enzymatic assay kits (KHB Company, Shanghai, China) following the manufacturer's instructions.

Protein expression and purification

To express recombinant proteins, expression plasmids were transformed into *E. coli* BL21 (DE3) PlysS cells (TransGen Biotech, Beijing, China). Recombinant protein expression was induced using 1 mmol/L isopropyl-beta-thiogalactopyranoside (IPTG) at 20 °C overnight. After centrifugation at 4500 × *g* for 10 min at room temperature, *E. coli* cells were suspended in Tris-buffered saline (pH 7.4) and disrupted with a lysis buffer (BR0005, ACE Biotechnology, Nanjing, China) on ice. After centrifugation at 8000 × *g* for 10 min at 4 °C, the homogenates were purified using a $Ni2^+$-NTA Sepharose (GE Healthcare, NJ, USA) affinity column and eluted with a buffer containing 50 mmol/L Tris pH 8.0, 500 mmol/L NaCl, and 300 mmol/L imidazole. The eluted proteins were then dialyzed overnight at 4 °C in 1 L dialysis buffer containing 10 mmol/L Tris-HCl (pH 7.5), 300 mmol/L NaCl, 1 mmol/L phenylmethylsulfonyl fluoride, and 1 mmol/L beta-mercaptoethanol. Finally, the proteins were concentrated with an Amicon ultra centrifugal filter (MWCO = 10 kD, Millipore, MA, USA) to

about 2 mg/ml and aliquoted into small fractions. The aliquots were flash-frozen in liquid nitrogen and transferred to −80 °C for long-term storage.

Plasmid construction

The mouse *Fgf6* and *Fgfbp1* promoters were amplified from mouse genomic DNA and subcloned into a pGL3-basic luciferase reporter vector (Promega, WI, USA). The mutant *Fgf6*-luciferase reporter with a deletion at the putative negative GR-binding site was generated using a Mut Express II Fast Mutagenesis Kit (C214, Vazyme, Nanjing, China). The fragment of *Fgfbp1* promoter with a deletion at the putative ATF3-binding site was generated by gene synthesis (BioSune, Shanghai, China), and subcloned into pGL3-basic luciferase reporter vector luciferase reporter plasmid. The primers used in this experiment are listed in **Additional file 1: Table S2**. All eukaryotic and prokaryotic expression vectors were purchased from the Public Protein/Plasmid Library (Geneppl, Nanjing, China).

Phase separation assay

For live cell imaging, AML12 cells expressing FGFBP1-EGFP or FGF5-mCherry were cultured in glass bottom dishes (D35C4-20-1-N, Cellvis, CA, USA) overnight to reach the desired density. Live cell imaging was performed on a Leica TCS SP8 confocal microscopy system at 37 °C in a live-cell imaging chamber using a 63 × oil objective and processed with Leica's LAS X and ImageJ software. For fluorescence recovery after photobleaching (FRAP) assay, the FGFBP1-EGFP and FGF5-mCherry condensates were photobleached using a laser intensity of 100% at 480 nm and 561 nm, respectively. Recovery was recorded for the indicated time. Images were processed and analyzed using ImageJ software and values are given relative to pre-bleach time points.

References

1. Fitzsimons CP, Van Hooijdonk LW, Schouten M, Zalachoras I, Brinks V, Zheng T, *et al.* Knockdown of the glucocorticoid receptor alters functional integration of newborn neurons in the adult hippocampus and impairs fear-motivated behavior. Mol Psychiatry. 2013;18(9):993-1005.
2. Liu C, Zhou B, Meng M, Zhao W, Wang D, Yuan Y, *et al.* FOXA3 induction under endoplasmic reticulum stress contributes to non-alcoholic fatty liver disease. J Hepatol. 2021;75(1):150-62.
3. Chen Y, Chen L, Wu X, Zhao Y, Wang Y, Jiang D, *et al.* Acute liver steatosis translationally

controls the epigenetic regulator MIER1 to promote liver regeneration in a study with male mice. Nat Commun. 2023;14(1):1521.

4. Gonçalves DA, Silveira WA, Manfredi LH, Graça FA, Armani A, Bertaggia E*, et al.* Insulin/IGF1 signalling mediates the effects of β(2) -adrenergic agonist on muscle proteostasis and growth. J Cachexia Sarcopenia Muscle. 2019;10(2):455-75.

5. Schmidt EK, Clavarino G, Ceppi M, Pierre P. SUnSET, a nonradioactive method to monitor protein synthesis. Nat Methods. 2009;6(4):275-7.

Table S1 Patient demographics and clinical characteristics

**Variable**	**ELISA-Q (*n* = 19)**	**ELISA-BDL (*n* = 14)**
Age (years, mean ± SD)	52.3 ± 14.3	49.2 ± 10.8
Sex [female, *n* (%)]	12 (63.2)	10 (71.4)
ALI etiology [*n* (%)]		
Viral	12 (63.2)	5 (35.7)
Drug	3 (15.8)	3 (21.4)
Cholestasis	4 (21.0)	5 (35.7)
AIH	0 (0.0)	1 (7.1)
Serum FGFBP1 (pg/ml, mean ± SD)	71.505 ± 64.249	-
|Slope| of ALT decline (mean ± SD)	1.972 ± 1.254	0.630 ± 0.346
|Slope| of AST decline (mean ± SD)	1.716 ± 1.366	0.559 ± 0.421

"-" indicates no data. *ALI* acute liver injury, *AIH* autoimmune hepatitis, *FGFBP1* fibroblast growth factor binding protein 1, *ALT* alanine aminotransferase, *AST* aspartate aminotransferase, *ELISA-Q* enzyme-linked immunosorbent assay-quantifiable, *ELISA-BDL* enzyme-linked immunosorbent assay-below detection limit, *SD* standard deviation

Table S2 The primers used in this study

Gene	Sequence (5' – 3')
qRT-PCR	
Fgf6-RT-F	CTGTACACAACGCCCAGCTT
Fgf6-RT-R	TTGTTTGGAAGGAGGGTTTCTC
Nr3c1-RT-F	CCGGGTCCCCAGGTAAAGA
Nr3c1-RT-R	TGTCCGGTAAAATAAGAGGCTTG
Fgfbp1-RT-F	CTAACCTCAAGCTGGTGAACCCC
Fgfbp1-RT-R	TCTCTAATGGCCATGGTCTGGGT
Vtn-RT-F	CCCCTGAGGCCCTTTTTCATA
Vtn-RT-R	CAAAGCTCGTCACACTGACA
Dkk3-RT-F	TGAGGCAGTGGCTACACAAG
Dkk3-RT-R	GCTGGTATGGGGTTGAGAGA
Atf3-RT-F	TTTTCCGGGAGTTTCATCAGA
Atf3-RT-R	CACTTTCCACCATGGCAGATC
18s-F	AGTCCCTGCCCTTTGTACACA
18s-R	CGATCCGAGGGCCTCACTA
36b4-F	TCCAGGCTTTGGGCATCA
36b4-R	ATCAGCTGCACATCACTCAGA
Gapdh-F	AGGTCGGTGTGAACGGATTTG
Gapdh-R	TGTAGACCATGTAGTTGAGGTCA
ChIP-qPCR	
p*Fgf6*-nGRE-F	CACCATCCATCACCTGCCAT
p*Fgf6*-nGRE-R	GGGATTCTGAGAGCCACTGT
p*Fgf6*-nGRE Adjacent-F	TGAGAGAAGGGGAGAAGGGG
p*Fgf6*-nGRE Adjacent-R	GCCGCCTCCAAGAGTTATGT
p*Fgfbp1*-ATF3-F	TCGAAGAGCCAGGCAAACC
p*Fgfbp1*-ATF3-R	AACTGGGAGTGGGAGCTAGT
p*Fgfbp1*-ATF3 Adjacent-F	ACTAGCTCCCACTCCCAGTT

Gene	Sequence (5’ – 3’)
p*Fgfbp1*-ATF3 Adjacent-R	AGGCTGCTATAGTGTTTGTCCC
Clone PCR	
p*Fgf6*-F	CGTGCTAGCCCGGGCTCGAGATCCCATGTTCAATCCCCGG
p*Fgf6*-R	CCGGAATGCCAAGCTTCCATCCACCTTGCCTCTCAG
p*Fgf6*-ΔnGRE-F	CAGCCAGCTCCACCTTTTCGCAGAGATGCTCT
p*Fgf6*-ΔnGRE-R	AGAGCATCTCTGCGAAAAGGTGGAGCTGGCTG
p*Fgfbp1*-F	CGTGCTAGCCCGGGCTCGAGCTCAGAAGCTCACCACTGGA
p*Fgfbp1*-R	CCGGAATGCCAAGCTTCAGGTTGGAGGGAAGCCTAG

qRT-PCR quantitative real-time PCR, *ChIP-qPCR* chromatin immunoprecipitation quantitative PCR, *Fgf6* fibroblast growth factor 6, *Nr3c1* nuclear receptor subfamily 3 group c member 1, *Fgfbp1* fibroblast growth factor binding protein 1, *Vtn* vitronectin, *Dkk3* dickkopf WNT signaling pathway inhibitor 3, *Atf3* activating transcription factor 3, *18s* 18S ribosomal RNA, *36b4* ribosomal protein lateral stalk subunit P0, *Gapdh* glyceraldehyde-3-phosphate dehydrogenase

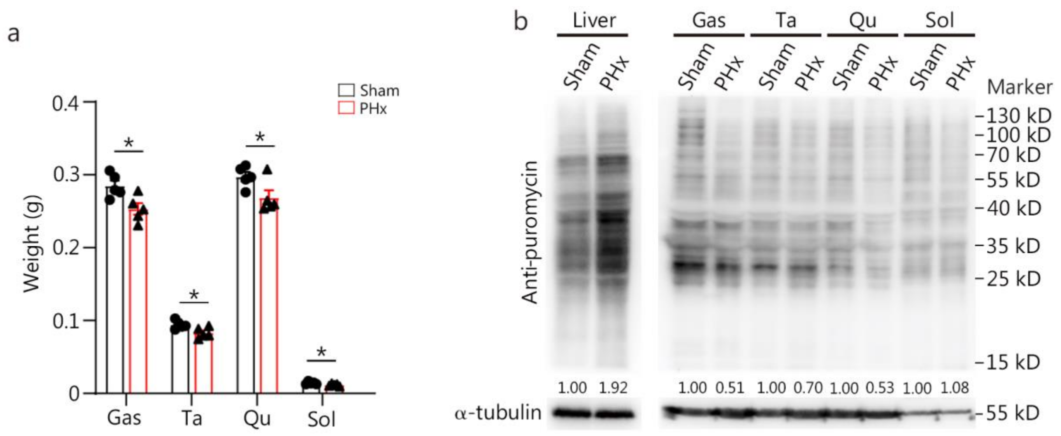

Fig. S1 Tissue weight and Western blotting analysis of the protein synthesis rate of mice 12 h after PHx or sham surgery. **a** Tissue weight in the gastrocnemius (Gas), tibialis anterior (Ta), quadriceps (Qu), and soleus (Sol) of sham and PHx mice (n = 5). **b** Images of the Western blotting, tracked with SUnSET analysis, showing changes in protein synthesis rate in liver, Gas, Ta, Qu, and Sol of sham and PHx mice. The levels of puromycin relative to the α-tubulin were shown through quantification, with the level of puromycin in the sham setting as 1.00. $^{*}P < 0.05$. PHx partial (2/3) hepatectomy, SUnSET surface sensing of translation

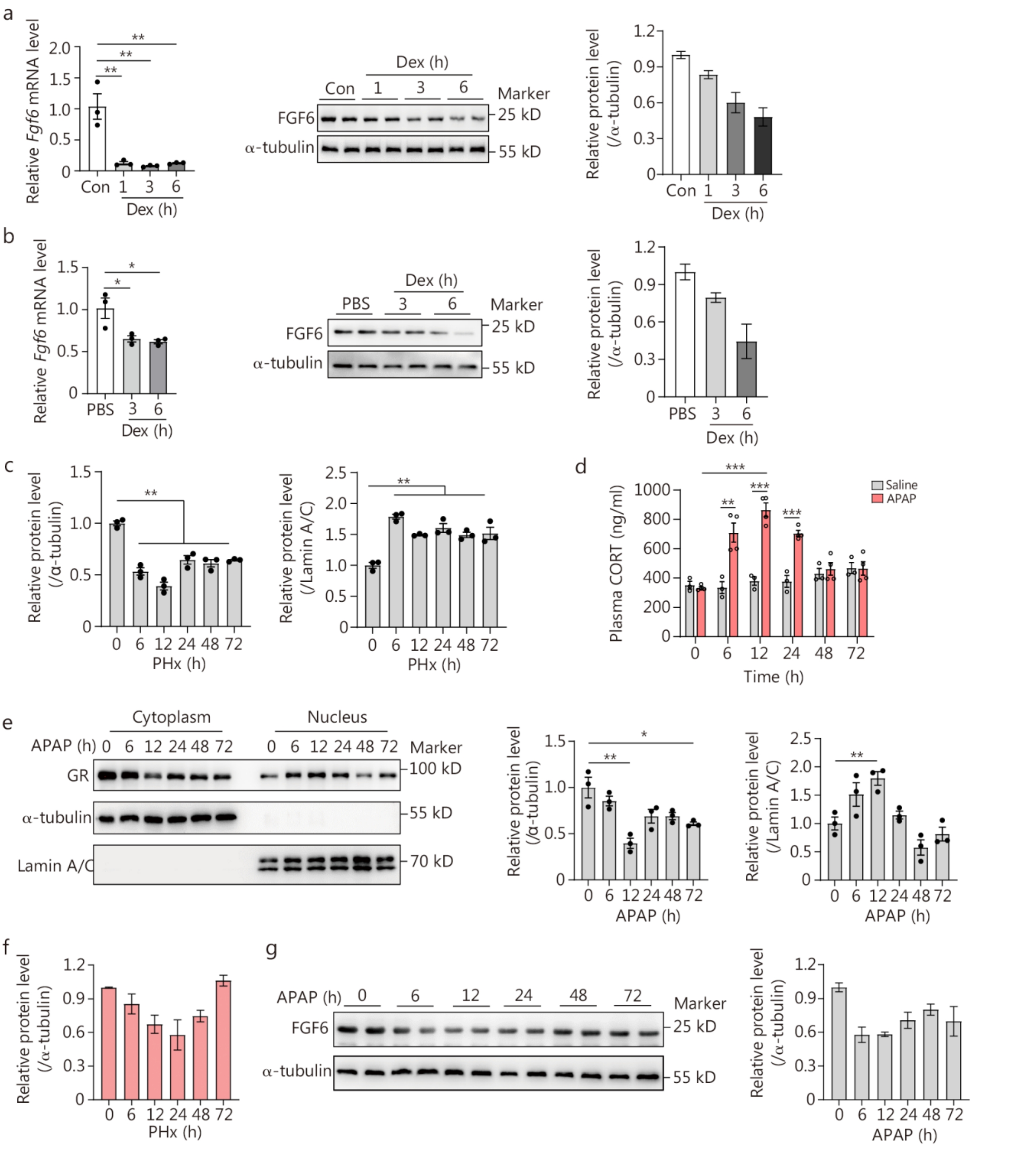

Fig. S2 Dexamethasone (Dex) treatment inhibits fibroblast growth factor 6 (FGF6) expression, and acetaminophen (APAP)-induced acute liver injury activates GCs signaling transduction. **a** Differentiated primary myoblast treated with Dex (10 μmol/L), the mRNA (n = 3) and protein level of *Fgf6* (n = 2) were detected at the indicated times. **b** Eight-week-old C57BL/6 mice were injected with Dex (100 μg/kg) or PBS, and the mRNA (n = 3) and protein level of *Fgf6* (n = 2) were detected at the indicated times. **c** Quantification of GR protein levels in the cytoplasm and nucleus of Gas at the

indicated times after PHx (Fig. 1j). **d** Plasma concentration of corticosterone (CORT) over time post-APAP dosing (n = 5). **e** GR protein levels in the cytoplasm and nucleus of the gastrocnemius (Gas) at the indicated times after APAP dosing with quantification (n = 3 independent biological replicates). **f** Quantification of FGF6 protein levels in the Gas at the indicated times after PHx (Fig. 1k). **g** FGF6 protein levels and their corresponding quantification in the Gas at the indicated times after APAP dosing (n = 2). $^{*}P < 0.05$, $^{**}P < 0.01$, $^{***}P < 0.001$. GCs glucocorticoids, GR glucocorticoid receptor, PBS phosphate-buffered saline

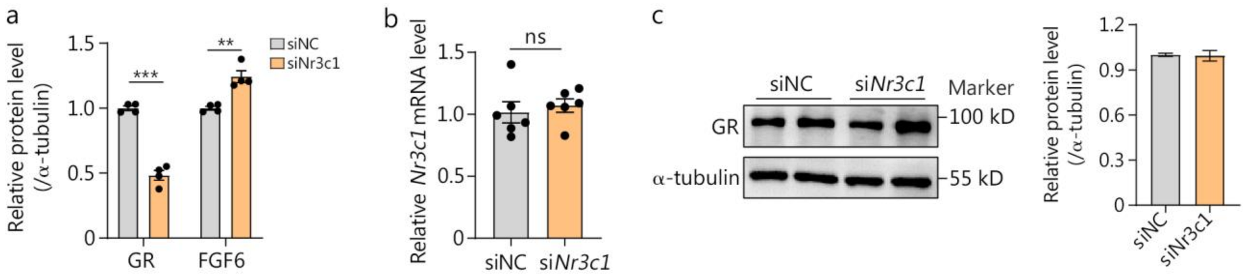

Fig. S3 Skeletal muscle specific-delivery of si*Nr3c1* has no effect on the level of GR in the liver. **a** Quantification of GR and FGF6 in the Gas of mice 24 h after PHx (Fig. 2c). **b** mRNA expression levels of *Nr3c1* in liver (n = 6). **c** Protein levels of GR in the liver with quantification (n = 2). Data are presented as the mean ± standard error of the mean (SEM). ns non-significant, GR glucocorticoid receptor, ns non-significant, Nr3c1 nuclear receptor subfamily 3 group c member 1

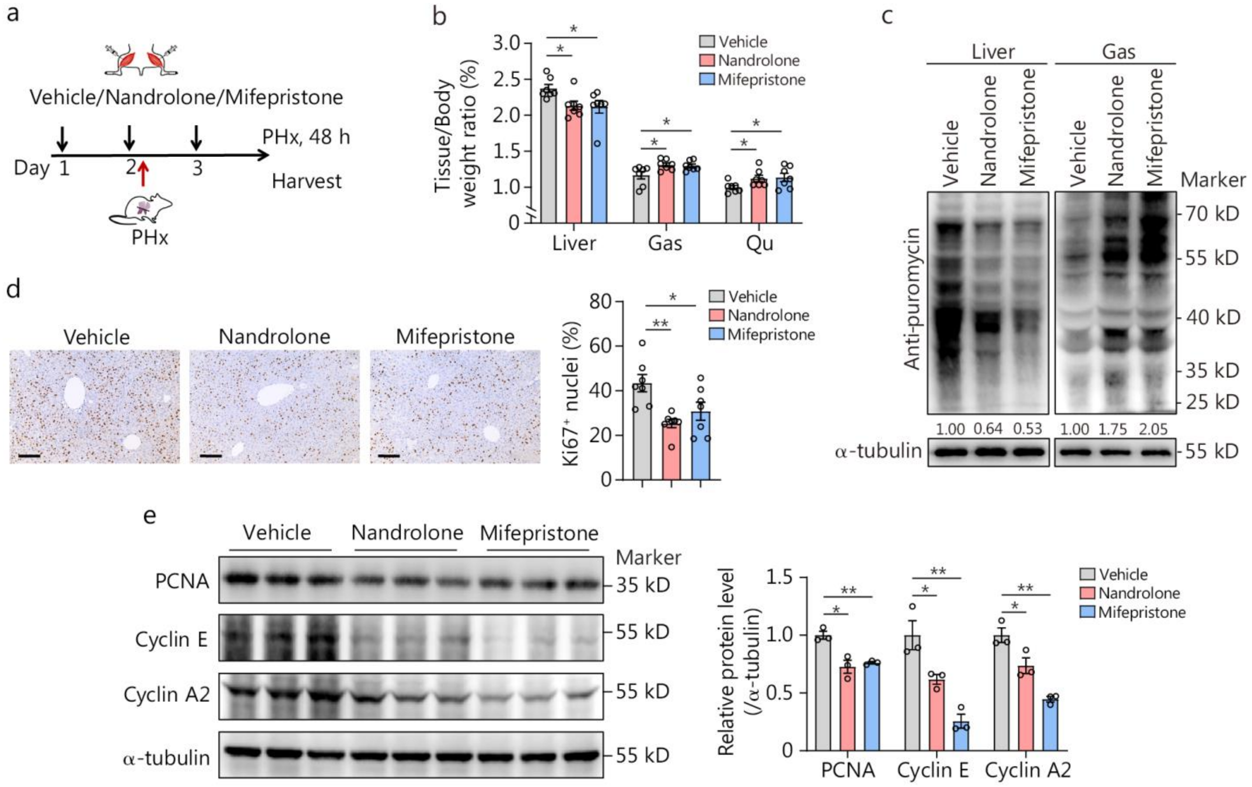

Fig. S4 Skeletal muscle administration of nandrolone or mifepristone impaired liver regeneration. **a** Schematic of partial (2/3) hepatectomy (PHx) surgery in 8-week-old male C57BL/6J mice, where the gastrocnemius (Gas) and tibia anterior (Ta) muscles were intramuscularly administered vehicle, nandrolone, or mifepristone for 3 times. **b** Tissues [including liver, gas, and quadriceps (Qu)]/Body weight ratios determined at 48 h after PHx (n = 7). **c** Western blotting analysis of the protein synthesis rates in the liver and Gas of vehicle, nandrolone, and mifepristone mice 48 h after PHx (n = 3 independent biological replicates). **d** Representative liver Ki67 immunohistochemistry (for indicating the proliferating cells) results and quantification of Ki67^{+} nuclei (n = 7). Scale bar = 100 µm. **e** Western blotting analysis of hepatic proliferation-related proteins proliferating cell nuclear antigen (PCNA), cyclin E, and cyclin A2 expression with the corresponding quantification (n = 3). $^{*}P < 0.05$, $^{**}P < 0.01$

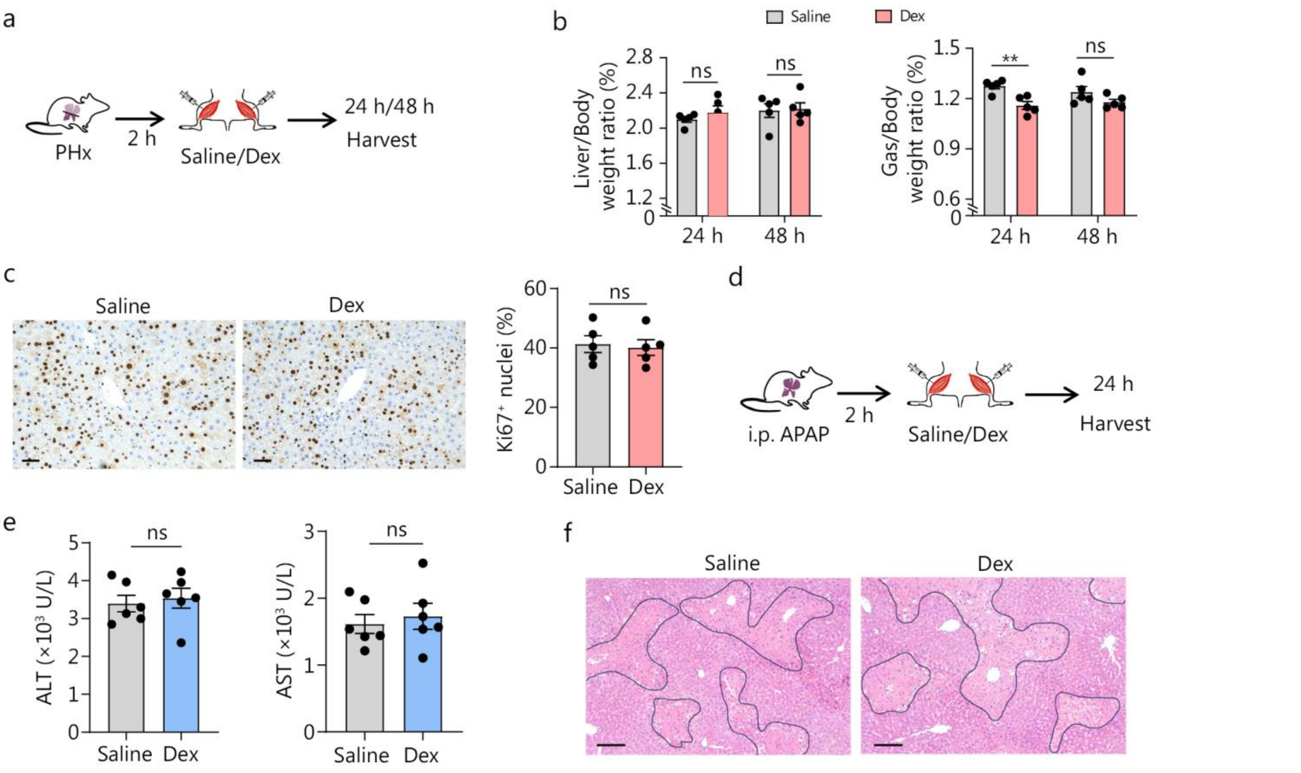

Fig. S5 Intramuscularly administered low-dose dexamethasone (Dex) has no effect on liver regeneration after acute liver injury (ALI). **a** Schematic of partial (2/3) hepatectomy (PHx) surgery in 8-week-old male C57BL/6J mice, where the gastrocnemius (Gas) and tibialis anterior (Ta) muscles were intramuscularly administered low-dose Dex (50 μg/kg) 2 h post-PHx. **b** Liver/Body weight and Gas/Body weight ratios determined at the 24 and 48 h after PHx ($n = 5$). **c** Representative liver Ki67 immunohistochemistry results and quantification of $Ki67^+$ nuclei at 48 h after PHx ($n = 5$). Scale bar = 25 μm. **d** Schematic of intraperitoneal (i.p.) injection of APAP (300 mg/kg) in 8-week-old male C57BL/6J mice, where the Gas and Ta muscles were intramuscularly administered low-dose Dex (50 μg/kg) 2 h post-APAP dosing. **e** Serum concentrations of ALT and AST ($n = 6$). **f** Representative liver H&E (necrotic areas circled with black lines) results. Scale bar = 100 μm. $^{**}P < 0.01$. ns non-significant, APAP acetaminophen, H&E hematoxylin and eosin, ALT alanine aminotransferase, AST aspartate aminotransferase

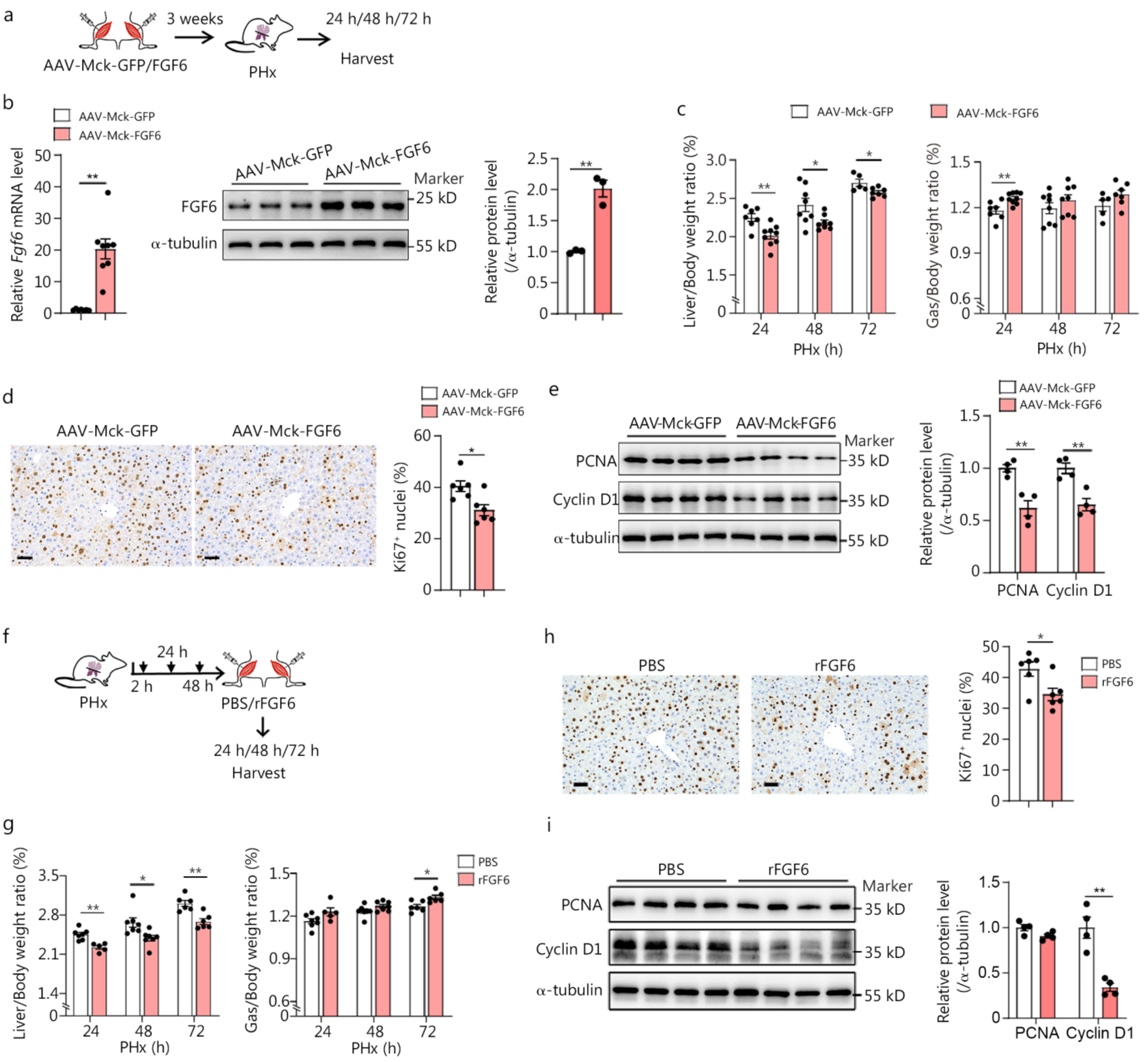

Fig. S6 Skeletal muscle overexpression of FGF6 inhibits liver regeneration after PHx in mice. **a** Schematic of the animal experiments. AAV-Mck-FGF6 or AAV-Mck-GFP were administered to the Gas and Ta depots of 8-week-old C57BL/6 mice. PHx was performed 3 weeks later, and the livers and Gas were analyzed 24, 48, and 72 h post-PHx. **b** The mRNA (n = 8) and protein levels of FGF6 in Gas at the 24 h after PHx (n = 3). **c** Liver/Body weight and Gas/Body weight ratios determined at 24, 48, and 72 h after PHx (n = 5 – 9). **d** Representative liver Ki67 immunohistochemistry results and quantification of Ki67$^+$ nuclei at 48 h after PHx (n = 6). Scale bar = 50 μm. **e** Western blotting analysis of hepatic proliferation-related proteins PCNA and cyclin D1 expression with the corresponding quantification at48 h after PHx (n = 4). **f** Schematic of the animal experiments. rFGF6 (10 μg/kg) or PBS were administered to the Gas and Ta depots 2, 24, and 48 h after PHx of 8-week-old C57BL/6

mice, and the livers and Gas were analyzed 24, 48, and 72 h post-PHx. **g** Liver/Body weight and Gas/Body weight ratios determined at 24, 48, and 72 h after PHx ($n = 5 – 7$). **h** Representative liver Ki67 immunohistochemistry results and quantification of Ki67$^+$ nuclei at 48 h after PHx ($n = 6$). Scale bar = 50 μm. **i** Western blotting analysis of hepatic proliferation-related proteins PCNA and cyclin D1 expression with the corresponding quantification at 48 h after PHx ($n = 4$). The data (**b**) were log-transformed and then analyzed using an unpaired Student's *t*-test. $^*P < 0.05$, $^{**}P < 0.01$. AAV adenovirus-associated virus, Mck muscle creatine kinase, GFP green fluorescent protein, FGF6 fibroblast growth factor 6, Gas gastrocnemius, PHx partial (2/3) hepatectomy, PCNA proliferating cell nuclear antigen, rFGF6 recombinant FGF6 protein, PBS phosphate-buffered saline

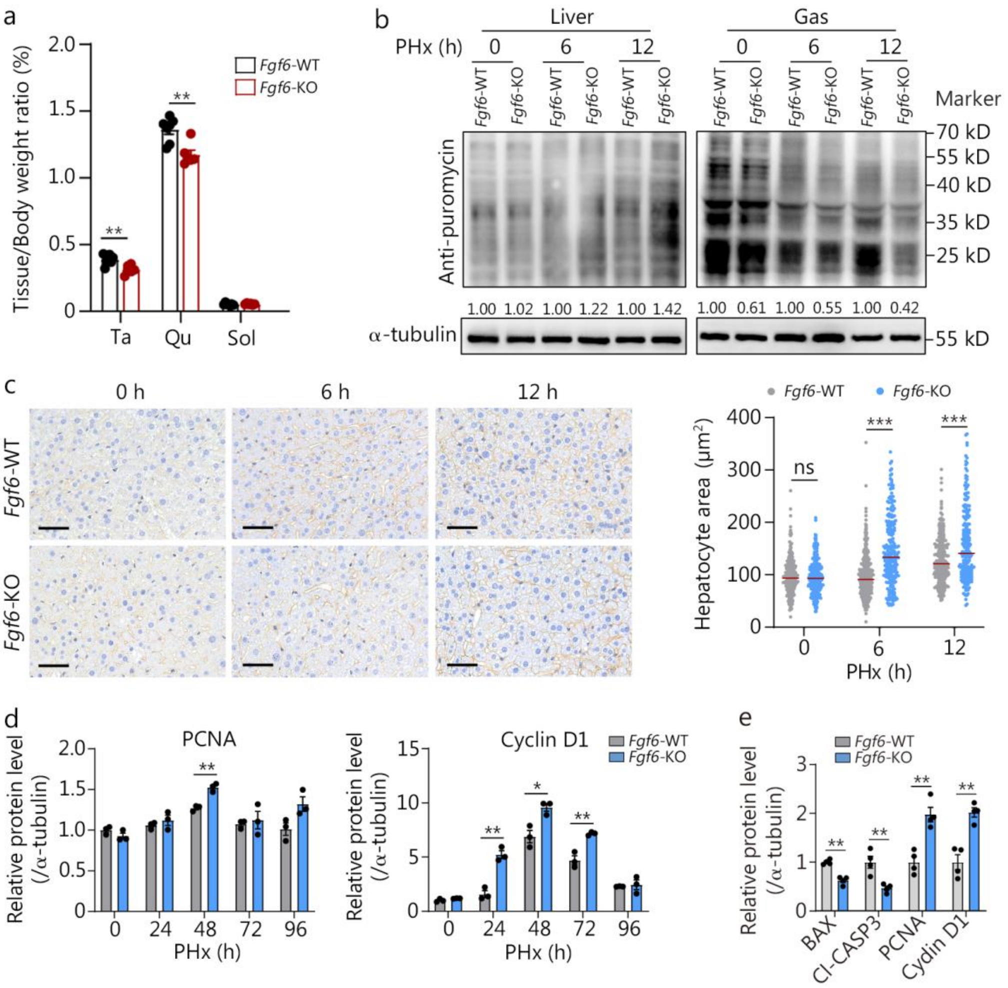

Fig. S7 *Fgf6* deficiency promotes liver regeneration after PHx. **a** Tissue weight as a percentage of body weight in the muscle of mice 24 h after PHx ($n = 6$). **b** Western blotting analysis of the protein synthesis rates in the liver and Gas of *Fgf6*-WT and -KO mice 0, 6, and 12 h after PHx ($n = 3$ independent biological replicates). The levels of puromycin relative to the α-tubulin were shown through quantification, with the level of puromycin in the WT setting as 1.00. **c** Representative liver β-catenin immunohistochemistry results and quantification of the hepatocyte area ($n = 3$). Scale bar = 50 μm. **d** Quantification of the hepatic proliferation-related proteins PCNA and cyclin D1 (G1/S-specific) at the indicated time points after PHx (Fig. 3d). **e** Quantification of hepatic apoptosis and proliferation-related proteins BAX, Cl-CASP3, PCNA, and cyclin D1 expression (Fig. 3h). $^{**}P < 0.01$, $^{***}P < 0.001$. ns non-significant, PHx partial (2/3) hepatectomy, Fgf6 fibroblast growth factor 6, WT wild-type, KO knockout, Gas gastrocnemius, Ta tibialis anterior, Qu quadriceps, Sol soleus

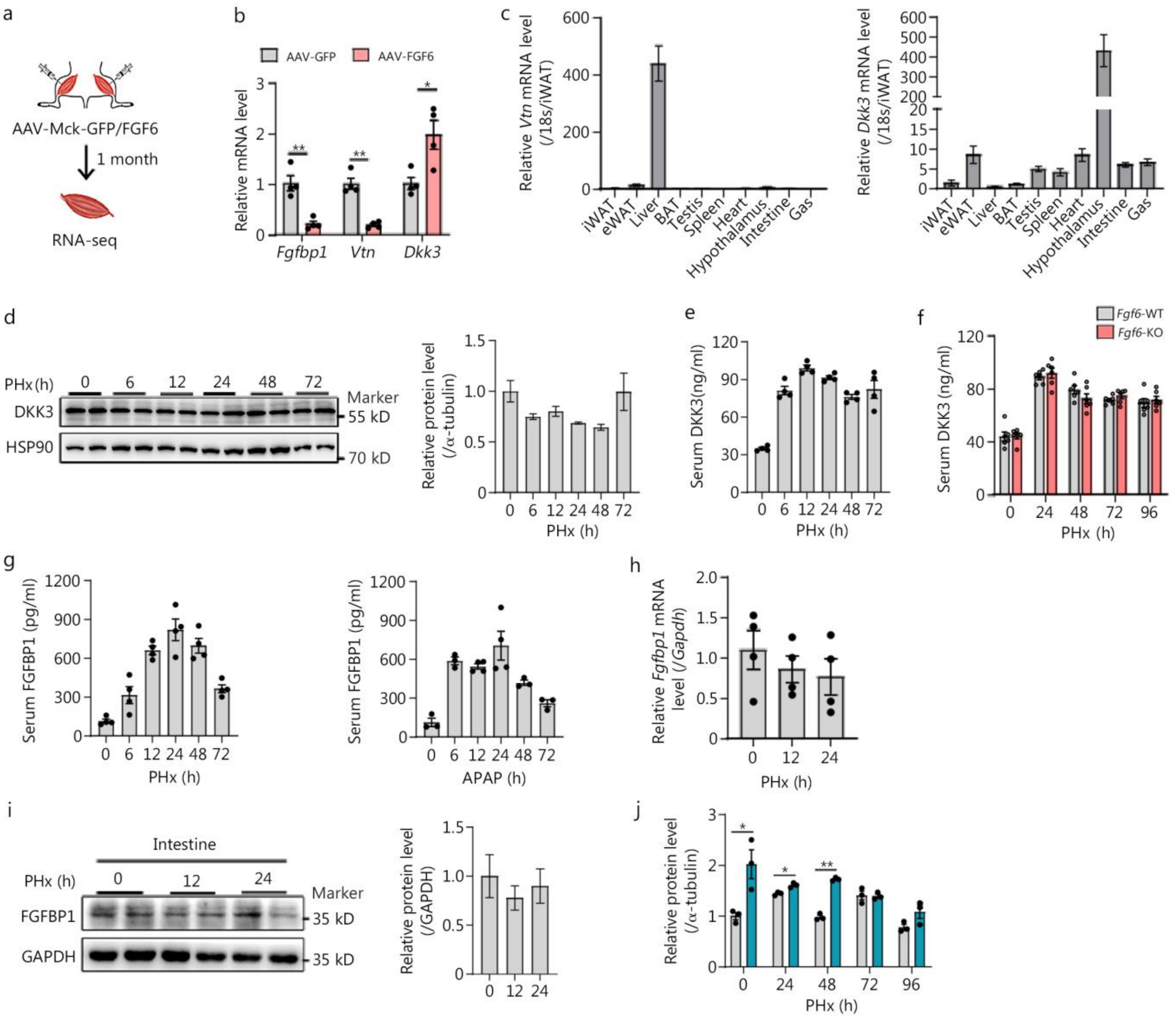

Fig. S8 Determination of the FGF6 targets secretory factors in Gas muscles. **a** Schematic of the experimental setup. **b** qRT-PCR verified the mRNA expression of 3 top genes ($n = 4$). **c** The mRNA expression patterns of *Vtn* and *Dkk3* ($n = 5$). **d** DKK3 protein levels with quantification in the Gas at the indicated times after PHx ($n = 2$). **e** Serum concentration of DKK3 at the indicated times after PHx ($n = 4$). **f** Serum concentrations of DKK3 in the *Fgf6*-WT and -KO mice at the indicated time points after PHx ($n = 6$). **g** Serum concentration of FGFBP1 at the indicated times after PHx and APAP dosing ($n = 4$). **h** qRT-PCR analysis of *Fgfbp1* mRNA expression in intestine after PHx ($n = 4$). **i** FGFBP1 protein levels in the intestine at the indicated times after PHx ($n = 2$). **j** Quantification of FGFBP1 protein levels in the Gas of *Fgf6*-WT and -KO mice at the indicated times after PHx (Fig. 5c). $^{*}P < 0.05$, $^{**}P < 0.01$. FGF6 fibroblast growth factor 6, Gas gastrocnemius, qRT-PCR quantitative real-time PCR, DKK3 dickkopf WNT signaling pathway inhibitor 3, FGFBP1 fibroblast growth factor binding

protein 1, PHx partial (2/3) hepatectomy, AAV adenovirus-associated virus, GFP green fluorescent protein, Vtn vitronectin, iWAT inguinal white adipose tissue, eWAT epididymal white adipose tissue, BAT brown adipose tissue

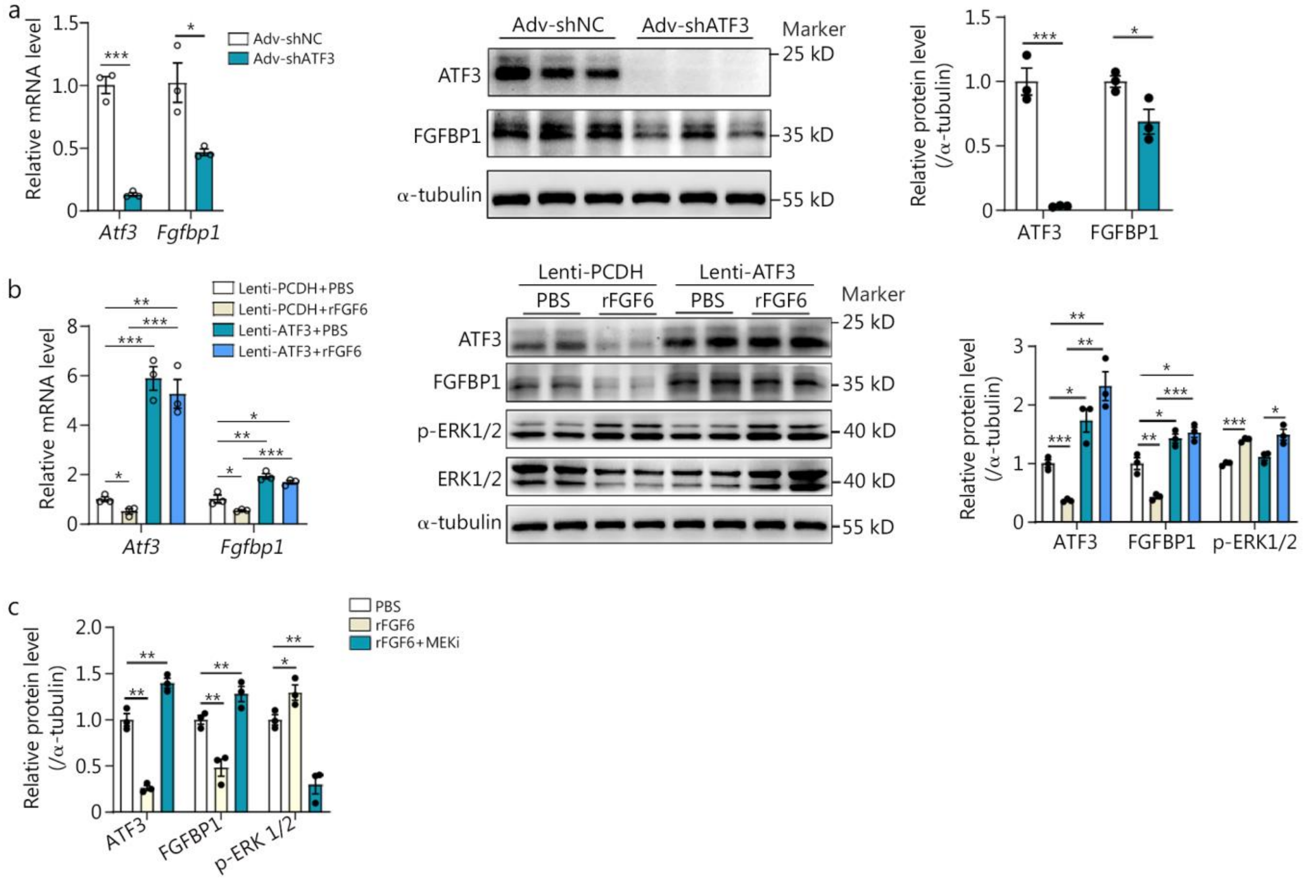

Fig. S9 FGF6 suppresses the transcription of *Fgfbp1* via ERK-ATF3 axis in muscle. **a** Knockdown of *Atf3* decreased the expression of FGFBP1 in differentiated C2C12 cell lines ($n = 3$). **b** Overexpression of ATF3 rescued the expression of FGFBP1 in differentiated C2C12 cell lines ($n = 3$). **c** Quantification of ATF3, FGFBP1 and p-ERK1/2 in protein levels in Fig. 5i. $^{*}P < 0.05$, $^{**}P < 0.01$, $^{***}P < 0.001$. FGF6 fibroblast growth factor 6, ERK extracellular signal regulated kinases, ATF3 activating transcription factor 3, FGFBP1 fibroblast growth factor binding protein 1, rFGF6 recombinant FGF6 protein, PCDH plasmid cloning and delivery vector with hygromycin, PBS phosphate-buffered saline

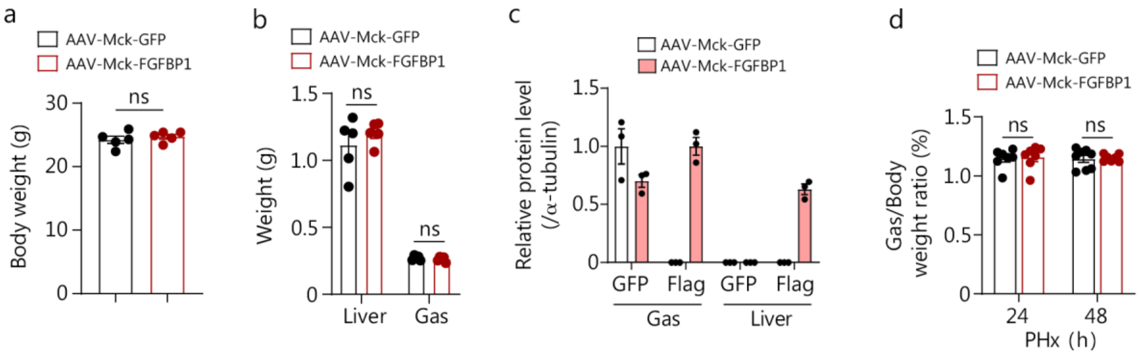

Fig. S10 The effects of skeletal muscle injection with AAV-Mck-FGFBP1 or AAV-Mck-GFP. **a** No changes in body weight (n = 5). **b** No changes in liver and Gas weight (n = 5). **c** Quantification of GFP and Flag protein levels in the Gas and liver (Fig. 5l). **d** Gas/Body weight ratio determined at 24 and 48 h after PHx (n = 7). ns non-significant, Gas gastrocnemius, FGFBP1 fibroblast growth factor binding protein 1, PHx partial (2/3) hepatectomy, AAV adenovirus-associated virus, Mck muscle creatine kinase, GFP green fluorescent protein

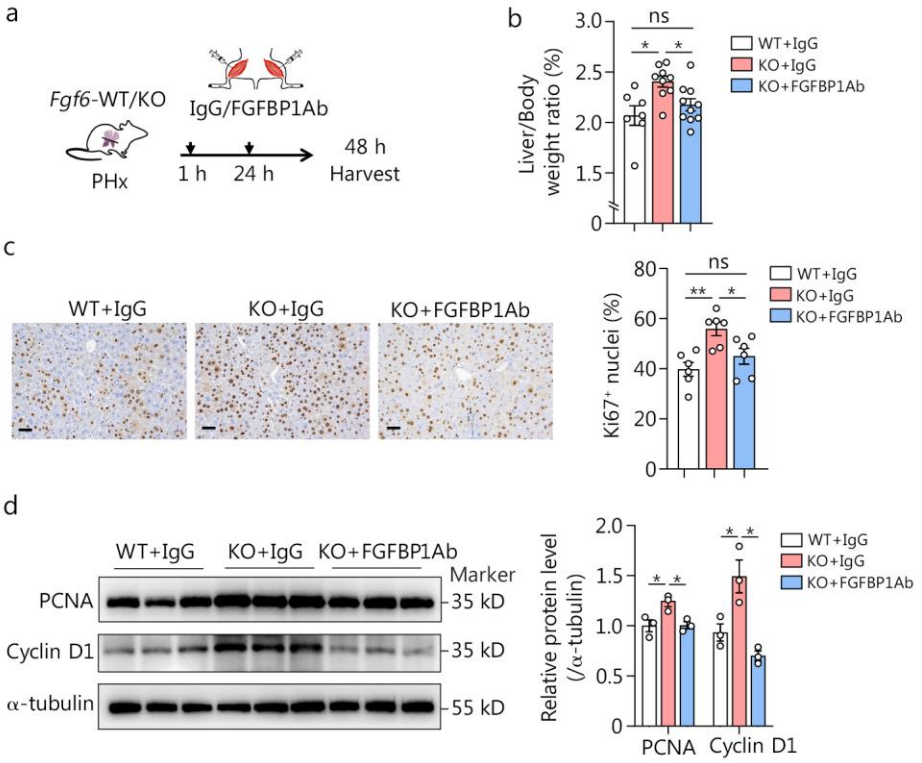

Fig. S11 Blocking FGFBP1 specifically in the skeletal muscle of *Fgf6*-KO mice abolished the protective effects in these mice upon PHx. **a** Schematic of FGFBP1Ab or IgG (0.5 mg/kg body weight) administration in the Gas and Ta depots of *Fgf6*-WT and -KO mice after PHx. **b** Liver/Body weight ratio (n = 7 – 10). **c** Representative liver Ki67 immunohistochemistry results and quantification of Ki67$^+$ nuclei (n = 6). Scale bar = 50 μm. **d** Western blotting analysis of hepatic proliferation-related proteins PCNA and cyclin D1 expression with the corresponding quantification (n = 3). $^*P < 0.05$, $^{**}P < 0.01$. ns non-significant, FGFBP1 fibroblast growth factor binding protein 1, PHx partial (2/3) hepatectomy, PCNA proliferating cell nuclear antigen, WT wild-type, KO knockout, FGFBP1Ab FGFBP1-neutralizing antibody

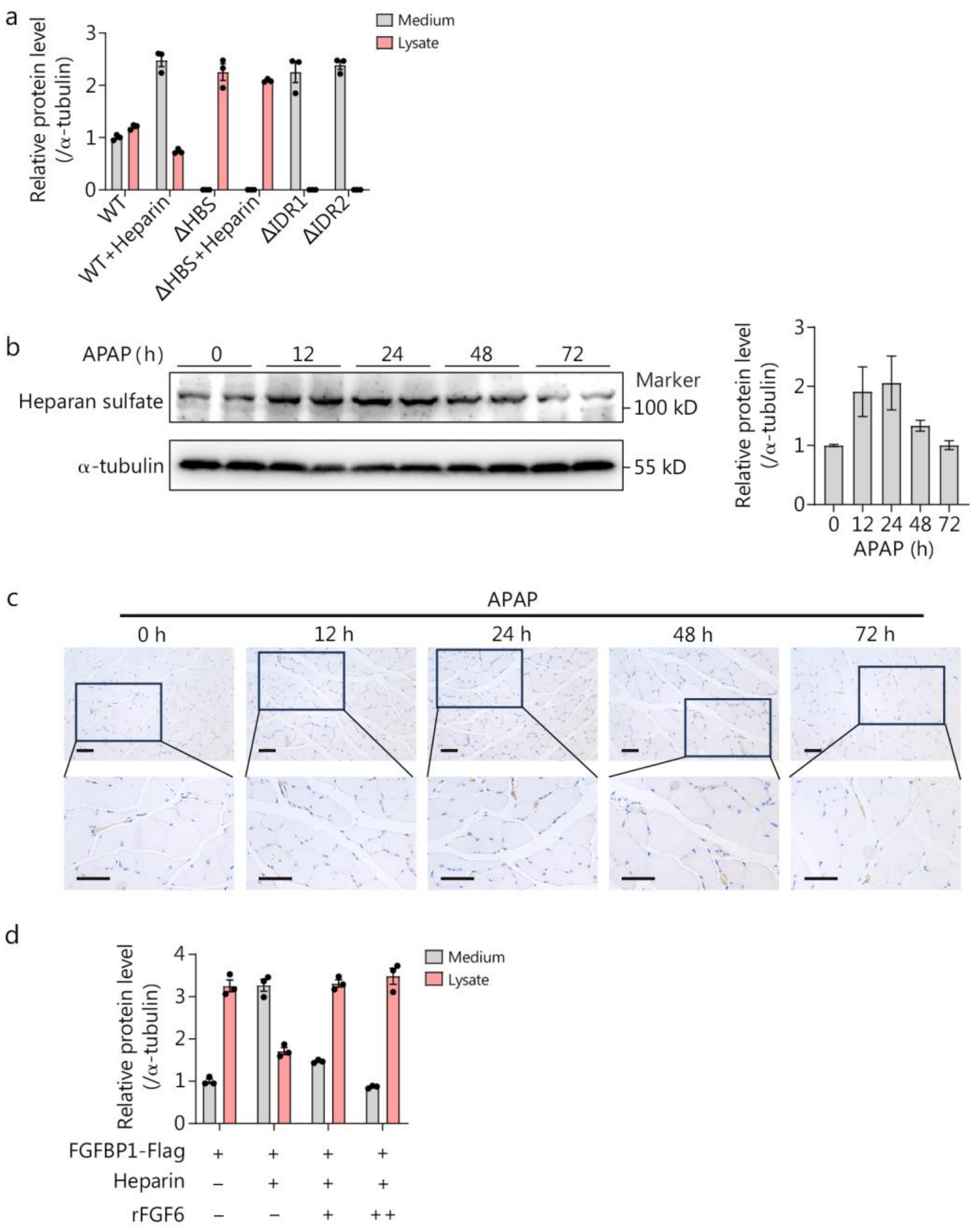

Fig. S12 Dynamic changes of heparan sulfate in muscle after acute liver injury (ALI). **a** Quantification of Flag levels in the cell culture medium and cell lysate (Fig. 6g). **b** Western blotting analysis of heparan sulfate in Gas at the indicated times after APAP dose with the corresponding quantification (n = 2). **c** Representative heparan sulfate immunohistochemistry of mouse Gas sections at the indicated times after APAP dose. Scale bar = 50 μm. **d** Quantification of Flag levels in the cell culture medium and cell lysate (Fig. 6i). Gas gastrocnemius, APAP acetaminophen

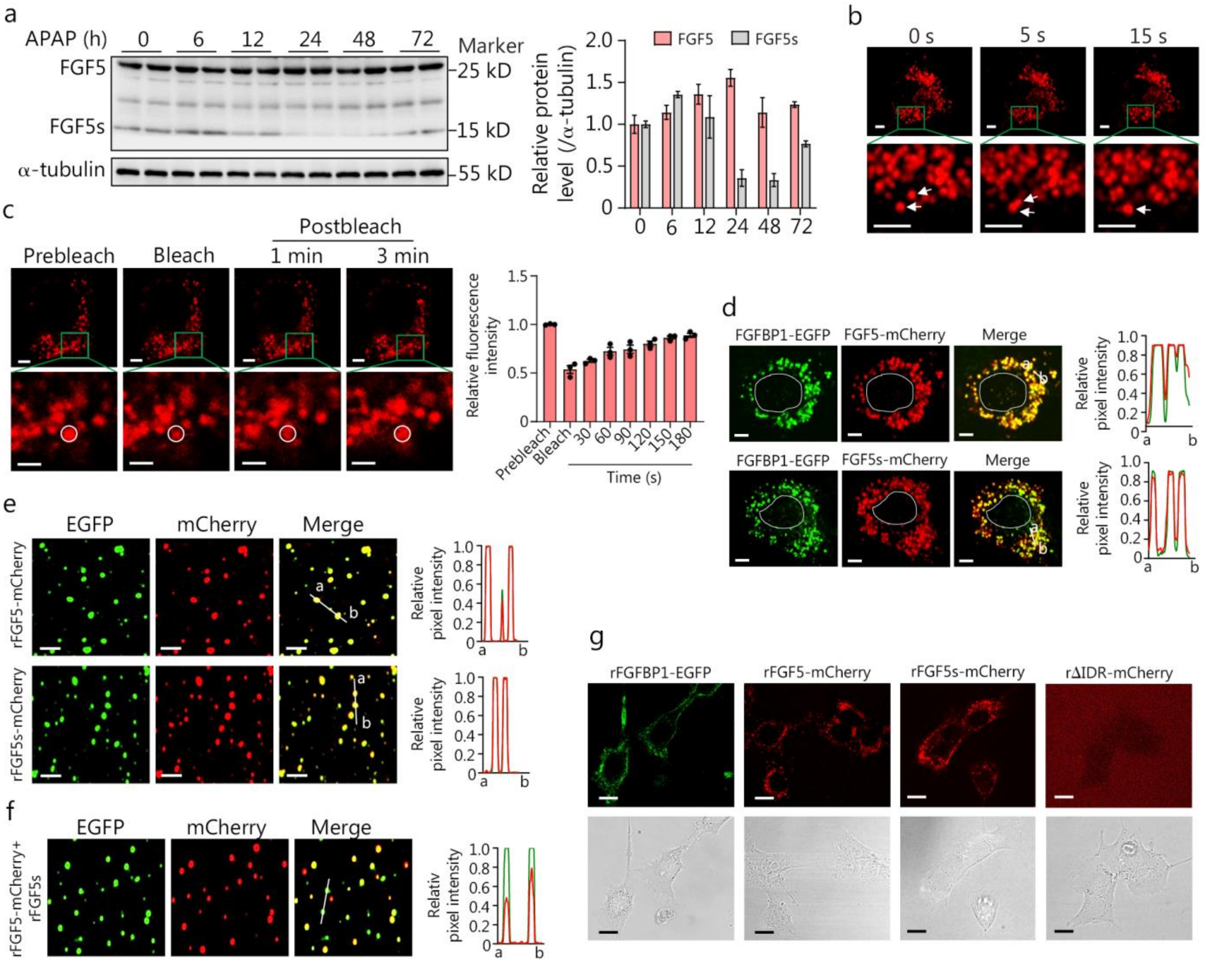

Fig. S13 FGF5 undergoes LLPS and colocalization with FGFBP1 condensates. **a** Hepatic protein levels of FGF5 and FGF5s at the indicated times after APAP dosing, with the corresponding quantification (n = 2). **b** Representative images of the fusion of FGF5-mCherry condensates (arrowheads) over time in AML12 cells expressing FGF5-mCherry. Scale bar = 5 μm. **c** Representative micrographs of FGF5-mCherry condensates before and after photobleaching. FRAP quantification of FGF5-mCherry condensates over a period of 3 min. Scale bar = 2.5 μm. **d** Images of colocalization assay in AML12 cells co-expressing FGFBP1-EGFP with FGF5-mCherry or FGF5s-mCherry via confocal microscopy and representative curves describing the distribution of the relative fluorescence intensities of FGFBP1 (green) and FGF5 or FGF5s (red). Scale bar = 5 μm. **e** Images of purified protein colocalization assay in vitro via confocal microscopy (rFGFBP1-EGFP, 2 μmol/L; rFGF5-mCherry, 5 μmol/L; rFGF5s-mCherry, 3 μmol/L) and representative curves describing the distribution of the relative fluorescence intensities of FGFBP1 (green) and FGF5 or FGF5s (red). **f** Confocal microscopy images of the colocalization of rFGFBP1-EGFP (2 μmol/L) and rFGF5-mCherry (5 μmol/L) mixed with rFGF5s (5 μmol/L) and representative curves describing the distribution of the relative

fluorescence intensities of FGFBP1 (green) and FGF5 (red). **g** Images of purified protein forming droplets on the cell surface via confocal microscopy. rFGFBP1-EGFP (5 nmol/L), rFGF5-mCherry (20 nmol/L), rFGF5s-mCherry (10 nmol/L), rΔIDR-mCherry (10 nmol/L). Scale bar = 10 μm. FGF5 fibroblast growth factor 5, LLPS liquid-liquid phase separation, FGFBP1 fibroblast growth factor binding protein 1, APAP acetaminophen, FRAP fluorescence recovery after photobleaching, EGFP enhanced green fluorescent protein, FGF5s short form of FGF5

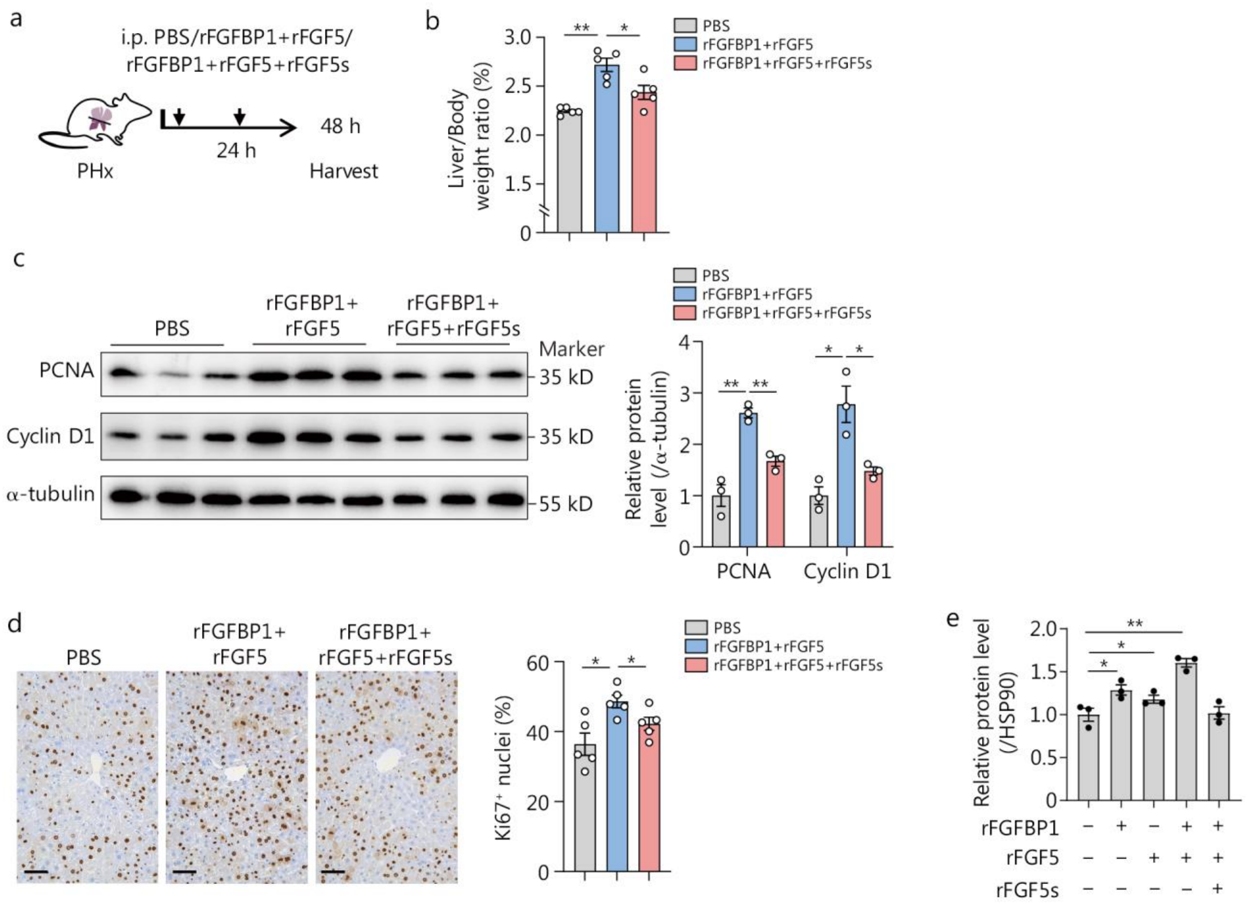

Fig. S14 FGFBP1, FGF5, and FGF5s combination therapy on liver regeneration of acute liver injury (ALI) mice. **a** Schematic of administering the indicated recombinant protein or PBS in PHx mice. **b** Liver/Body weight ratio (n = 5). **c** Western blotting analysis of hepatic proliferation-related proteins PCNA and cyclin D1 expression with the corresponding quantification (n = 3). **d** Representative liver Ki67 immunohistochemistry results, and quantification of Ki67$^+$ nuclei (n = 5). Scale bar = 50 μm. **e** Quantification of p-ERK expression in AML12 cells treated with the indicated recombinant proteins (Fig. 8g). $^*P < 0.05$, $^{**}P < 0.01$. FGFBP1 fibroblast growth factor binding protein 1, FGF5 fibroblast growth factor 5, FGF5s short form of FGF5, PHx partial (2/3) hepatectomy, PCNA proliferating cell nuclear antigen, rFGFBP1 recombinant FGFBP1 protein, PBS phosphate-buffered saline, rFGF5 recombinant FGF5 protein, rFGF5s recombinant FGF5s protein

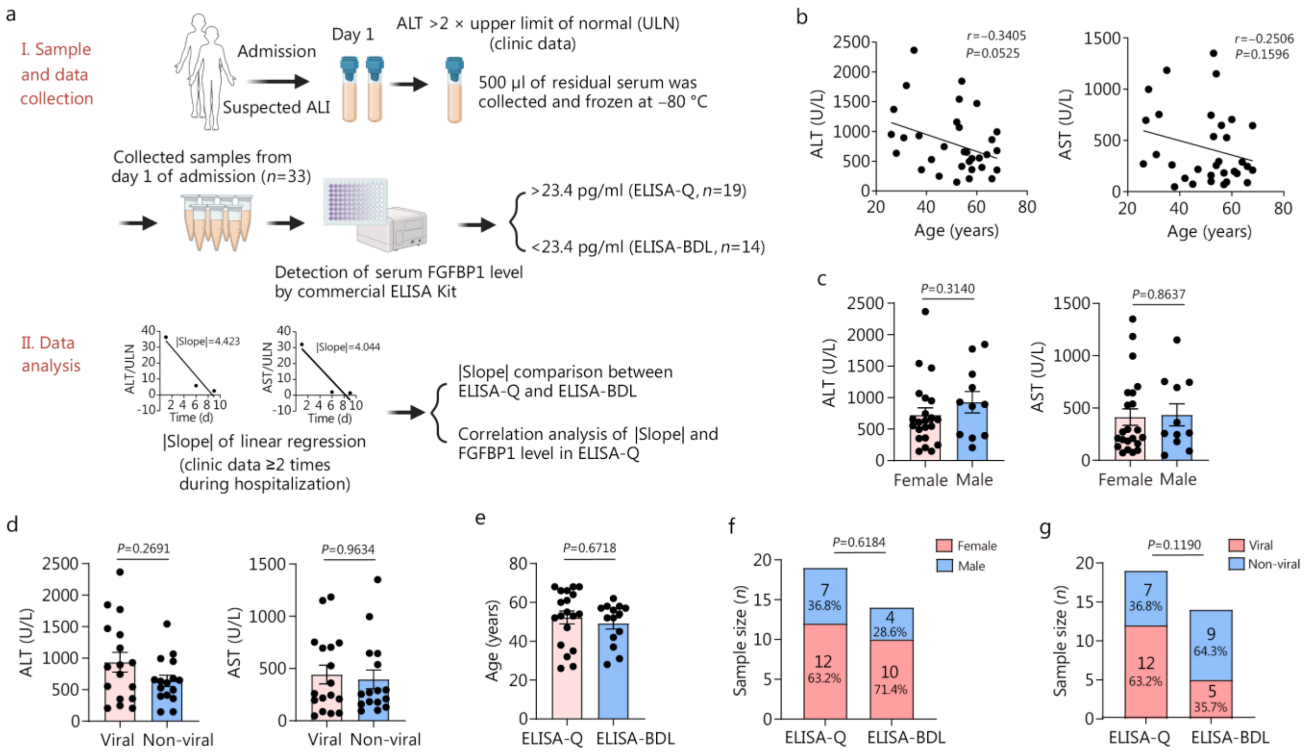

Fig. S15 Clinical characteristics of patients with acute liver injury (ALI). **a** Schematic of the clinical samples and data collection and analysis. Image created with BioRender.com. **b** The correlation between initial serum ALT or AST to age (n = 33). **c** The initial ALT and AST levels were compared between the groups of female and male patients (n = 22 and 11, respectively). **d** Patients were divided into two groups: the viral ALI group and the non-viral ALI group (n = 17 and 16, respectively). The initial ALT and AST levels were compared between the two groups. **e** Comparison of age between the groups of ELISA-Q and ELISA-BDL (n = 19 and 14, respectively). **f** Analysis of sex distribution between the groups of ELISA-Q and ELISA-BDL (n = 19 and 14, respectively). **g** Analysis of the proportion of viral and non-viral ALI in groups of ELISA-Q and ELISA-BDL (n = 19 and 14, respectively). FGFBP1 fibroblast growth factor binding protein 1, ALT alanine aminotransferase, AST aspartate aminotransferase, ELISA-Q enzyme-linked immunosorbent assay-quantifiable, ELISA-BDL enzyme-linked immunosorbent assay-below detection limit
